# An IoT-based smart emotion recognition system by using internal body parameters

**DOI:** 10.1038/s41598-026-35982-9

**Published:** 2026-02-04

**Authors:** Tayyaba Rashid, Imran Sarwar Bajwa, Jungsuk Kim

**Affiliations:** 1 The Islamia University of Bahawalpur, Bahawalpur, Pakistan; 2 Gachon University, Seongnam-si, South Korea

**Keywords:** Emotion recognition, Internet of Things (IoT), Machine learning, Internal body parameters, Smart emotion recognition system (SERS), Computational biology and bioinformatics, Engineering, Health care, Mathematics and computing

## Abstract

Emotion recognition using physiological signals has gained significant attention in recent years due to its potential applications in mental health monitoring, human–computer interaction, and stress management. This study focuses on recognizing six emotional states neutral, happy, sad, fear, anger, and surprise using internal body parameters such as blood pressure, oxygen saturation, blood glucose, heart rate, and body temperature. Leveraging an Internet of Things (IoT) enabled framework, real-time data was collected from participants. An exhaustive experimental assessment has been performed on 11 different classification algorithms of the machine learning platform. Among the algorithms, the Random Forest algorithm performed better than all other algorithms with 90.56% accuracy and 93.34% F1-score. Moreover, the precision and recall of the proposed system are extremely high. Model Robustness and generalization performances were evaluated by conducting internal as well as external validation. On conducting internal validation through k-fold cross-checking, the accuracy increased to 93.18%, clearly validating the consistency in the performance of the model. Further, the external validation was conducted by using the conventional DEAP emotional tasks, showing a collective accuracy of about 94% along with very good max and weighted average precision, recall, and F1-score values for all classes of emotions. This clearly validates the efficacy of the chosen physiological features as well as the correctness of the devised approach. The findings indicate that physiological signals, combined with IoT and machine learning, provide an effective framework for emotion recognition. This research contributes to the development of real-time, non-invasive emotion recognition systems, with promising applications in healthcare, wearable devices, and personalized user experiences. Future work will explore the integration of additional physiological parameters and advanced deep-learning models for enhanced accuracy and scalability, and usage in advanced technology.

## Introduction

Daily life of human’s is heavily influenced by emotions which have an impact on one’s decisions, behaviors, actions, social interactions, ideas, health, and well-being. Emotion recognition is a widely studies and significant field of research. Emotion recognition can be studied either using external body parameters or internal body parameters. Fields like emotion recognition by using gait analysis, facial emotion recognition, speech recognition, and analysis of body language typically use external body parameters as walking pattern, face expression, voice and body gesture. However, according to recent studies^1^ internal body parameters can provide deeper insights into human emotion as compared to external body parameters. Hence, there is a need to further study and investigate the features of internal body parameters and it’s role in emotion recognition.

Emotion recognition by the internal body parameters is a subdomain of affective computing. Affective computing is a multifaceted topic that focuses on developing systems and technologies that interpret, predict, and respond to human emotions and affective states. A person’s emotional state might be inferred by utilizing internal components by measuring physiological, neurological, and biological signals. In this article, we embark on a journey where emotions are recognized by internal body parameters such as heart rate variability, body temperature, blood pressure, the electrical signal of muscles, oxygen concentration, and blood Sugar.

The Internet of Things is a digital era of inter-connected objects that can converse with each other, and its services play a vital role in every aspect of modern life regarding health, business, homes, the agriculture industry, and many others^2^. Things that existed just in imagination and in our dreams such as digital assistants, smart environments, smart cars, smart healthcare systems, and smart homes, now can exist in real life with the help of IoT^3,4^. Internet of Things (IoT) can play an important role in Emotion recognition and it is a possibility that IoT can play a significant role in emotion recognition^5^. IoT provides the network infrastructure that collects, transmits, analyzes, and evaluates the data that is perceived from different sources, including sensors and devices^6^. Emotion Recognition can also play an important role in smart IoT applications as the integration of emotion recognition and IoT can improve performance and make it more responsive to human needs.

**Fig. 1:**
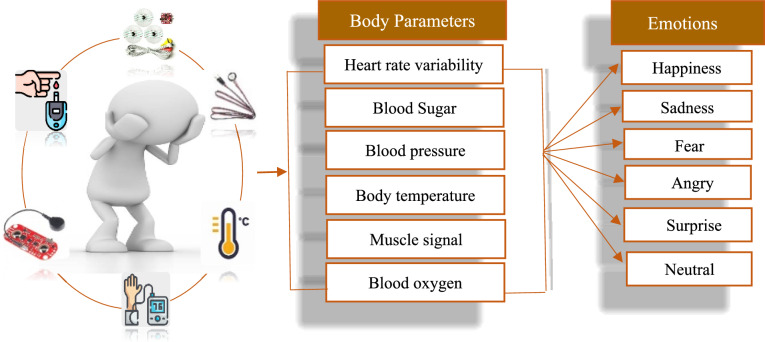
Human emotions interlinked with internal body parameters.

Previous research focused on external parameters for human emotion recognition. However, limited research is available that explores the potential of using internal body parameters to detect and predict human emotion. Moreover, the existing ßtheories use fewer body parameters for the prediction of human emotion.In this study, a novel emotion recognition system based on the processing of internal body parameters is presented. The proposed approach uses internal body parameters(Heart rate, oxygen concentration, blood pressure, blood glucose, body temperature, and electric signal of muscle) for precisely prediction of human emotions. The emotions are directly linked with the internally body parameters and it’s efficient way for prediction of emotions as it cannot be controlled or hide. It has been revealed that emotions significantly influence our physiological reactions, imprinting identifiable patterns and signals that are measurable and subject to analysis. In Contrast to existing theories, the proposed approach using more internal body parameters enabled by IoT for offering the robust system which may provide results more accurately. However, the proposed system focuses on six distinct emotions: Happy, sadness, anger, Fear, Neutral, and surprise. Figure 1 represents the relationship between the physiological signals and their respective emotion.

One of our motivation for designing an emotion recognition system is based on one of the SDGs(Sustainable Development Goals) goal of UNO that is Good health and well-being. In the field of mental health, continuous monitoring of internal signals can provide valuable perspectives on mood disorders, anxiety, and stress levels, facilitating personalized treatment and support.. An emotion recognition system incorporated in healthcare where elderly people are living alone, the patients require continuous monitoring of their health and emotional states, psychologists, Seafarers, long-distance drivers, and pilots can also benefit from the proposed system.

Most existing emotion recognition research is based on an emotion induction paradigm, where participants are first exposed to emotional stimuli, such as images, sounds, or videos, and physiological data are subsequently recorded. Although this methodology has been employed extensively, it may not capture naturally occurring real-time emotional fluctuations. There is a pressing need for systems to be developed to recognize emotions from physiological signals directly, without artificially inducing emotions. To overcome this limitation, the current study suggests an inverse method wherein inner body parameters are obtained first and afterward employed for determining the corresponding emotional state. Those systems can improve healthcare applications in real time, monitoring of mental well-being, and human–computer interaction.

To address the above challenges, the research aims to accomplish the following objectives:

- To design and develop an IoT-based smart Emotion recognition(SERS) that collects real-time values of Body Parameters (Body temperature, Blood sugar, Muscle signal, Heart rate, Oxygen Concentration, and Blood pressure) through interconnected sensors for continuous emotion monitoring.
- The dataset for the proposed methodology is evaluated for six different categories of emotions.
- To preprocess and analyze the gathered body parameter values to determine patterns relating to certain emotional states.
- To Implement and asses machine learning algorithms integrated with proposed (SERS) framework for analysis and interpretation of physiological parameters.
- To validate the performance of the proposed system using both real-time gathered data and the DEAP benchmark dataset.

The remainder of this article is organized as follows: Section II represents the Background & Related work and Section III presents the system model and design. Section IV outlines the Implementation of the system. The performance evaluation and experimental results are summarized in Section V and Section VI concludes the article and outlines future research directions.

## Background and related work

### Background

Emotions are intricate psychological and physiological states that encompass a broad spectrum of feelings, including happiness, sorrow, anger, fear, affection, and more. These emotions typically arise in response to internal or external stimuli, giving rise to a mix of physiological reactions, cognitive assessments, personal experiences, and behavioral responses.

The understanding and interpretation of human emotions have always been a fascinating and challenging endeavor. The traditional approaches to recognizing emotions rely on external cues such as vocal intonation, facial expression, body language, and gait Analysis. Emotion recognition by external cues can frequently manifest human emotions through various outward signs and these external factors offer insight information into a person’s feelings and emotions. External cues are explicitly important, they offer only a glance into the complex tapestry of human emotions. Emotions are not limited to external cues and what we display outwardly; they are also intimately intertwined into the very fabric of the human body.

In emotion recognition by external cues, Facial Emotion Recognition (FER) is one of the highly- studied, and widely used methods. Facial emotion recognition is a typical approach in which one can predict the emotional state of a person by his facial expressions. However, FER needs high-quality data of images and video so the zoning of the face can result in better output. In case, the data quality of image and video is not good, it’s difficult to predict the state of emotion. The one major drawback of FER is the unavailability of data as data is not provided due to privacy and security concerns. The other factor is that FER can be limited in accurately determining emotions due to conscious concealment of emotion.

Emotions can be recognized through speech by focusing on tonal features, and spoken language. Focusing on words, pitch, and tonal features, can interpret human emotions^7^. But many other factors affect the tonal features and pitch which are not relevant to emotions so the accuracy of this method is also affected. In Emotional recognition by speech, noise also affects predicting the emotional state accurately, so the removal of noise is also a time-consuming and expensive method.

**Fig. 2:**
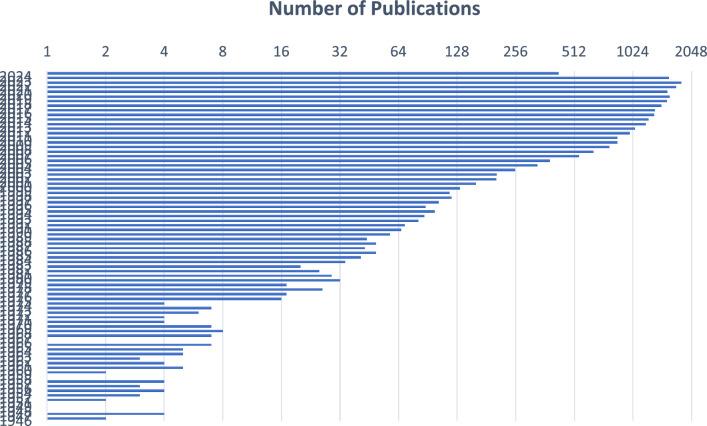
Total number of annual papers on emotion recognition.

One other method of emotion recognition is gait analysis. In gait analysis emotions are recognized by different positions and walking styles. Emotion recognition from gait focuses on walking patterns. A gait analysis study usually focuses on cadence, smart strides, and short double limbs. However, the gait analysis is not highly accurate in determining the emotional state as influenced by many factors. The emotion recognition by gait analysis depends on walking patterns and these factors can be changed due to other reasons such as fatigue, pain, injury, or change in footwear.

he field of study of ‘Emotion recognition’ is experiencing continuous growth and expansion with each passing day. As depicted in Fig. 2, there has been a steady increase in the number of publications per year in this field. Data from numerous published articles were gathered by utilizing the search term ‘Emotion recognition’ on the ‘PubMed’ website.

External cues have traditionally been the main ways through which emotions are expressed. However, emotions are not limited to external manifestation but emotions can be predicted accurately by internal body parameters as internal parameters are more strongly linked with emotions.

**Fig. 3:**
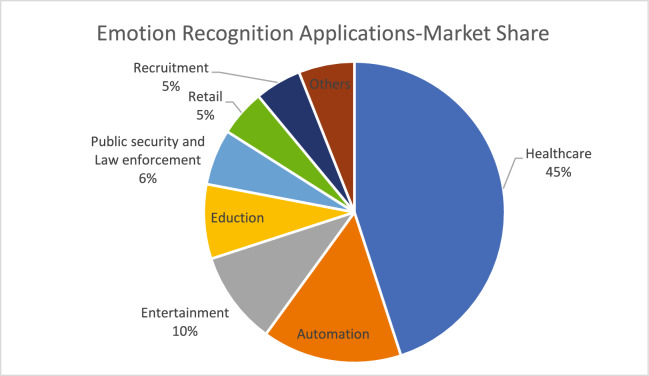
Global emotion detection and recognition market share by application.

Figure 3 depicts the emotion recognition application share in Market size.Emotion Recognition also plays a crucial role in smart IoT applications. The Integration of emotion recognition into IoT systems can boost IoT Intelligence and make it more responsive and robust to human needs. Some potential applications of emotion recognition with IoT are a healthcare monitoring system, Stress management system, Human Counselling System, Learning System, Smart homes, and Safety System. According to a report (Fortune Business Insight) the emotion recognition market size was €21billion in 2021 and it’s expected to grow €29 billion by 2029 because this technology can be used in every industry at different stages.

The IoT is a network of interrelated devices embedded with sensors, processors, and communication capabilities that allow self-monitoring, data exchange, and analysis without requiring human intervention. The IoT creates smart environments by bridging the physical world with digital platforms and makes these systems smarter, more effective, and highly automated. Applications area covers healthcare, smart homes, industries, transportation, and research where processing in real time enhances decision-making and responsiveness in systems^8^.

The IoT architecture enables a structured model connecting physical devices, communication networks, and intelligent applications. It also provides for smooth interaction between sensors, gateways, cloud servers, and end-user interfaces^8^. Though there are different models, most IoT systems are based on a multilayered structure that guarantees efficiency in data flow, processing, and security. The architecture design of layers makes IoT modular, scalable, and interoperable, addressing big applications like healthcare, smart cities, and emotion recognition systems.

**Fig. 4:**
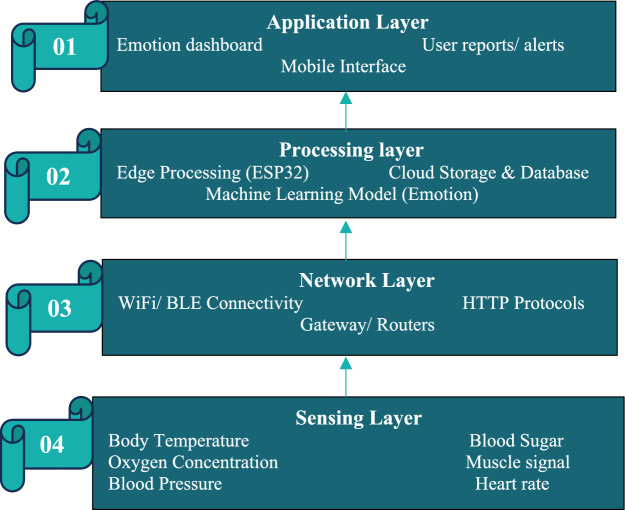
IoT Architecture for SERS (Smart Emotion Recognition).

The basic architecture of IoT includes four major layers: These are: (1) Sensing Layer, (2) Network Layer, (3) Processing Layer, and (4) Application Layer as described in Fig. 4. Each layer plays a specific role and is subject to different types of security threats, which have to be addressed for the reliability and trustworthiness of IoT systems^9^.

The sensing layer is the lowest layer in the IoT architecture and is responsible for sensing the physical world. Sensors and actuators make up this layer, where data on body temperature, blood pressure, heart rate, oxygen concentration, and muscle signals are collected. This layer converts real-world information into digital signals, having the responsibility of sending them to the next layer for processing. It forms the very base upon which the whole IoT system is built.

The network layer forwards the sensed data from devices to servers, cloud platforms, or edge gateways. The most common technologies used in this layer are Wi-Fi, Bluetooth and cellular networks (3G/4G/5G). Its major responsibility is ensuring that communications between IoT nodes and processing systems are secure, reliable, and fast. It stands between the physical environment and data analytics engines.

This Processing layer processes, analyzes, filters, and stores the data received. The processing layer carried out the task of data cleaning and machine learning. For an Emotion Recognition System, this layer applies algorithms to identify emotional states from internal body parameters. It ensures that only meaningful and refined information is forwarded to the application layer^9^.

The application layer delivers the services of IoT to the end users. It interprets the processed data and provides the results in a form that may be understandable, like dashboards or mobile apps, including alerts. In the smart emotion recognition system, this layer shows the detected emotions-such as happy, sad, fear, anger, etc.-and supports healthcare, smart monitoring, and assistive applications. It’s the interface between the system and the user.

Despite efficiency and quality of service offered by all layers, each layer in IoT architecture remains vulnerable to specific security risks due to distributed nature of devices, resource constraints, and open channels of communication. The vulnerabilities expose the system to various cyberattacks that might compromise the integrity, confidentiality, and availability of data. Some key security threats with their mitigation strategies are discussed for the respective layers of IoT in Table 1.

**Table 1:** Attacks on IoT layers and solutions.

Layers	Attacks	Solutions
Sensing	Sensor tempering, fake data injection	Tamper-proof sensors, anomaly detection
Network	Replay, DDos	Timestamps, firewalls
Processing	Cloud hijacking, malware	MFA, API security, encryption
Application	API misuse, credential theft	Strong password, HTTPs, access control

### Related work

Domínguez-Jiménez et al.^1^ proposed a method for emotion recognition through bio-signals. Their model predicts emotions using gloves that incorporate PPG and GSR sensors. The designed system identified three emotional states: amusement, sadness, and neutral. The author claims 100% accuracy for the prediction of emotion due to an intelligent model and by applying a support vector machine (SVM) for classification.

Ramzan et al.^10^ proposed a system that predicts emotion valence and arousal recognition by using physiological signals(EEG, ECG). In proposed system model was trained by inducing the emotions of 30 participants. Two feature vectors (EEG and ECG) were used to train the five different classifiers. EEG observation is used to train the classifier for valence recognition and ECG observation is used to train the classifier for arousal recognition.

Hassani, Bafdal et al.^11^ proposed a approach that predict three emotions(Anger, joy, Neutral). The experiment is done on three subjects. The author did not use machine learning algorithms for training and testing the data set due to fewer subjects and used Predictive and statistical analysis. Emotions are predicted by features of body temperature and GSR signals.

Santamaria et al.^12^ proposed a system that predicts emotion by using physiological signals such as ECG and GSR. The proposed approach predicts the valence and arousal level of emotions. The model was trained by using a Convolutional Neural Network (CNN). The system is validated by testing on the DEAP and MAHNOB datasets.

Kaur et al.^13^ proposed an approach of emotion recognition using IoT and physiological signals(ECG, body temperature). In the presented approach heart rate and body temperature values were collected and trained in the model by employing the KNN machine learning algorithm. The presented approach predicts the four types of emotion: happy, sad, relaxed, and angry.

Shu et al.^14^ proposed the approach that emotion is recognized using heart rate variability and wearable bracelets. The proposed work emphasizes the potential of using heart rate information obtained from wearable devices to identify emotions, with the goal of advancing consumer electronics designed for monitoring human emotions in everyday situations.

Susanto et al.^15^ The proposed approach recognized the emotion using the Galvanic skin response(GSR), which reveals the electric characteristics of human skin and identifies the presence of emotion. GSR signals are obtained by monitoring variations in skin resistance resulting from the activity of sweat glands in the skin. The proposed work crafted a deep hybrid neural network that incorporates a time-distributed 1D CNN and Residual Bidirectional GRU to capture more indicative features from the GSR signal.

Awais et al.^16^ presented IoT based approach that predicts four types (relaxing, boring, amusing, and scary) of emotion using physiological signals. In their proposed approach physiological signals (EMG, GSR, ECG, and SKT) collected from the human body and machine learning algorithms were employed for training the model.

Saffaryazdi et al.^17^ proposed approach that emotions are recognized by collecting the data with conversation. In their proposed approach an interviewer asks the participants to speak about the memories as much as possible and during conversation collect the physiological signals and predict the emotions. Their experiment conducted in temperature controlled room to minimize the effect of environment on physiological signals. A self-report questionnaire also filled by participants.

Han et al.^18^ proposed approach that predict the emotions by using Photoplethysmography and electromyography signals. The model trained by using deep learning. Their experiment was done on 32 participants (16 male and 16 female). There proposed approach predict the valence and arousal level of emotions and system validation done by DEAP dataset.

Al-Naji et al.^19^ used a system that utilized photoplethysmography (PPG), respiration rate, and oxygen saturation (SpO₂) to identify emotional states including calm, anxious, and excited. Their findings indicated the potential of using a combination of multiple physiological indicators for more reliable emotion detection.

Mano et al.^20^ proposed an IoT-based system for health smart homes that integrates patient identification and emotion recognition using physiological signals. Their system used cameras and wearable sensors to collect data, processed through machine learning algorithms to detect emotional states. The study demonstrated the feasibility of non-intrusive emotion monitoring in home environments, aiding in the early detection of emotional disorders.

Zhao et al.^21^ presented EmotionSense, a wearable wristband-based system that captures physiological signals (e.g., GSR and heart rate) and applies deep learning methods for emotion classification. The system demonstrated high accuracy in real-time emotion identification, utilizing IoT connectivity for information processing and storage. This solution emphasizes the effectiveness of integrating IoT with sophisticated machine learning for personalized emotion-aware solutions.

Tiwari et al.^22^ proposed an emotion recognition classification system with IoT, which combined EEG and other physiological signals. The scheme employed a two-stage instantaneous frequency-based filtering and correlation approach to process the EEG signals with strong emotion classification. The work highlights the capabilities of IoT in facilitating online data acquisition and processing, hence ideal for use in smart healthcare and human–machine interaction applications.

Shu et al.^23^ introduced a smart-bracelet-based wearable emotion recognition system based on heart rate data. The research used machine learning algorithms to analyze heart rate signals, which were found to yield high accuracy for the detection of emotions like happiness and sadness. The IoT architecture of the system permitted easy data transmission to a cloud server for real-time processing, proving the scalability of emotion recognition in healthcare environments.

Algarni et al.^24^ introduced an emotion recognition model based on deep learning for EEG signals with a Bi-directional Long Short-Term Memory (Bi-LSTM) network. Their method, tested using the DEAP dataset, attained high accuracy in emotional state classification of valence and arousal. The work exemplified the high potential of Bi-LSTM models for EEG-based emotion recognition and highlighted the need for efficient feature extraction and selection.

Middya et al.^25^ proposed a multimodal emotion recognition system based on deep learning with the fusion of audio and visual modalities at the model level. Their model using benchmark datasets such as SAVEE and RAVDESS provided high accuracy, proving that the fusion of multiple modalities has a much improved emotion recognition performance over single-modality systems.

Saganowski et al.^26^ performed a systematic review of emotion recognition with physiological signals from wearable devices in real-life situations. They reviewed 34 studies and found shared workflows comprising data acquisition, preprocessing, and machine learning for emotion classification. The research pointed out challenges including sensor reliability, imbalance in data, and absence of standard validation protocols, and accentuated the necessity of increased real-world, wearable-based emotion recognition studies.

Li et al.^27^ provide an exhaustive "tutorial and review" of EEG-based emotion recognition with both an introductory overview of the psychological and physiological roots of EEG signals that will be accessible to newcomers, as well as a structured overview of novel technical approaches adopted in recent years. They classify previous work based on signal acquisition, preprocessing, feature extraction, emotion modelling and classification, and then point out salient challenges like inter-subject variability, noise/artifact management, ecological validity and deployment in real-world gaps.

Houssein et al.^28^ gave an extensive overview of machine learning methods for EEG-based emotion recognition. The authors gave a summary of multiple datasets, preprocessing, and classification used in brain–computer interfaces. The authors highlighted the fact that EEG signals provide valid emotional information because of their direct association with brain activity. They also mentioned that deep learning methods have better recognition accuracy but issues like signal noise, inter-individual differences, and small datasets persist. In general, the paper pointed out the promise of EEG-based BCI systems blended with machine learning for emotion-aware health care and human–computer interactions.

Chaturvedi et al.^29^ provide a systematic overview of the use of physiological signals, including heart rate, galvanic skin response, blood volume pulse, and skin temperature, to infer emotional responses to music. They describe most salient sensor modalities, signal-processing methods, classification pipelines, and the experimental settings in which music-induced emotional states have been investigated. Their research points out that although music-mood recognition through wearables demonstrates encouraging outcomes, real-world deployments remain limited by factors such as inter-subject variation, context dependence of musical experience, and the predominance of laboratory-based experiments over naturalistic listening situations.

Lin et al.^30^ performed a thorough review on emotion judgment and recognition with physiological signals like EEG, ECG, EDA, and EMG. Their research compared different modalities of signals, explained their strengths and weaknesses, and pointed to challenges such as cross-subject variation, multimodal fusion, and environmental stability. Lin et al. highlighted that though physiological signals reveal meaningful aspects of human feelings, more research is required to make it more applicable in real-world scenarios and generalize the model.

Khare et al.^31^ carried out a decade-long systematic review of emotion recognition based on artificial intelligence methods. Their review considered various AI-based models used with physiological, facial, and speech data for emotion recognition. They pointed out the progress made through deep learning and multimodal fusion but reported that there were still ongoing challenges in the form of dataset diversity being sparse, there is no real-world testing, and ethical implications for emotion AI deployment. The authors also suggested future directions for enhancing transparency, generalization, and reliability in emotion recognition systems based on AI.

Cai et al.^32^ published an extensive review on emotion recognition systems based on different sensors, emotion models, approaches, and databases. They reviewed studies on various modalities including facial, audio, and physiological signals, noting that multimodal systems enhance accuracy but are subject to issues such as data synchronization and scalability. The authors called for standardized datasets and testing in real-world scenarios to further the validity of emotion recognition technologies.

Houssein et al.^33^ proposed a TFCNN-BiGRU model with a self-attention layer for self-adaptive emotion recognition from multi-channel EEG signals. Convolutional and recurrent neural networks are used in the model to extract spatial–temporal features and identify long-term dependencies, while the self-attention layer strengthens feature importance and suppresses noise. The experiments indicated that the proposed method outperformed previous methods in accuracy and robustness, illustrating its potential for emotion-aware applications in healthcare and human–computer interaction.

Sharma et al.^34^ have outlined a real-time hybrid face expression recognition model for emotion classification and prediction in the human resource domain. The model combines a deep convolutional neural network (DCNN) and the Haar cascade approach to accurately detect faces and examine emotions from real-time video streams. The system, which was trained on the FER dataset, showed high accuracy and reactivity for real-time emotion detection. The authors emphasized its ability to drive HR procedures like employee engagement and performance management alongside focusing on the need to resolve ethical and privacy issues that come with emotion-aware technologies.

Table 2 present the comparison of previous studies with the proposed work which display the parameters and purpose of study for emotion recognition.

i. Previous studies focused more on inducing emotions and then collect body parameters.
ii. Previous studies focused more on external cues for emotion recognition.
iii. Previous studies using fewer internal body parameters for emotion recognition.
iv. Intelligent decision making was not used in previous studies
v. Previous studies had not mention the range of body parameter values which indicate the respective emotions.

**Table 2:** Comparison of proposed work with most recent Studies of emotion recognition.

Study	Work year	Heart Rate	Body temperature	Skin conductance	Blood oxygen	Blood Sugar	EEG	EMG	Blood pressure	ECG
Relevant studies of Emotion recognition using physiological signals										
^35^	2016	✓	**×**	✓	**×**	**×**	**×**	**×**	**×**	**×**
^10^	2016	**×**	**×**	**×**	**×**	**×**	✓	**×**	**×**	✓
^11^	2017	**×**	✓	✓	**×**	**×**	**×**	**×**	**×**	**×**
^12^	2018	**×**	**×**	✓	**×**	**×**	**×**	**×**	**×**	✓
^36^	2018	✓	**×**	✓	✓	**×**	**×**	✓	**×**	✓
^14^	2020	✓	**×**	**×**	**×**	**×**	**×**	**×**	**×**	**×**
^1^	2020	✓	**×**	✓	✓	**×**	**×**	**×**	**×**	**×**
^17^	2022	✓	**×**	✓	✓	**×**	✓	**×**	**×**	**×**
^18^	2023	✓	**×**	**×**	✓	**×**	**×**	✓	**×**	**×**
Recent studies of Emotion recognition using physiological signals and IOT										
^13^	2018	**×**	✓	**×**	**×**	**×**	✓	**×**	**×**	**×**
^16^	2020	**×**	✓	✓	**×**	**×**	✓	✓	**×**	**×**
In Purposed system										
–		✓	✓	**×**	✓	✓	**×**	✓	✓	**×**

## Proposed architecture of SERS

Our proposed Smart Emotion Recognition system (SERS) used IoT approach for collecting Internal body parameters i.e. electric signal of muscle, electric signal of heart, body temperature, blood pressure, blood glucose, blood oxygen concentration and predict emotions on the basis of varying these internal body parameters by doing classification of machine learning algorithms. The proposed approaches shown in Fig. 2 that visually represented all the components, their interactions, and the operational strategy of the proposed Smart Emotion recognition system.

In the proposed system Data is collected from internal body parameters using the relevant sensors. Set up a continuous data collection process to capture real time internal body parameters reading. The data is collected by configure IoT devices to connect with the internal body parameter sensors(GSR, EMG, HRV, skin temperature, blood pressure, Spo2, glucose level, body temperature). A communication link is established between the sensors and IoT devices for transmitting the data securely.

**Fig. 5:**
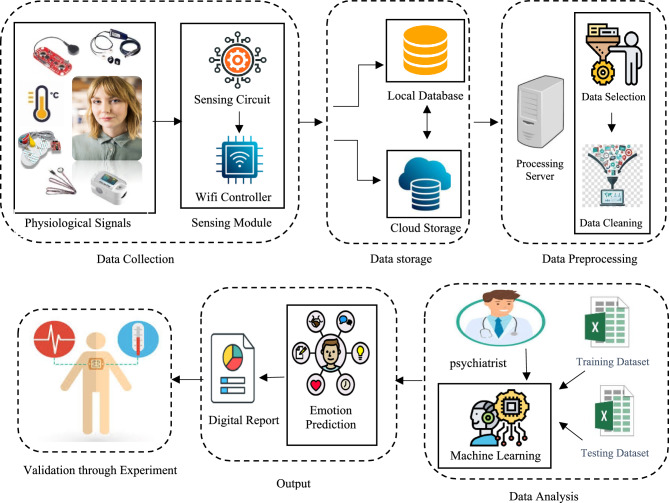
Proposed model for emotion recognition using IoT and machine learning.

Figure 5 represents the proposed model of emotion recognition. After Data Collection, the collected data is transmitted to both the cloud and the local database. The data is transmitted by using a Wi-Fi controller ESP32. ESP32 is a series of low-cost, low-power system-on-chip microcontrollers with integrated Wi-Fi and dual-mode Bluetooth. In the proposed system, after data storage the next phase is data preprocessing. The subsequent stage involves preprocessing the data to standardize the values within each interval. Process the raw data to mitigate noise, identify and handle outliers, and address missing values. Normalize the data to establish consistency across various internal body parameters. The normalized data for each interval is then stored as the dataset.

**Fig. 6:**
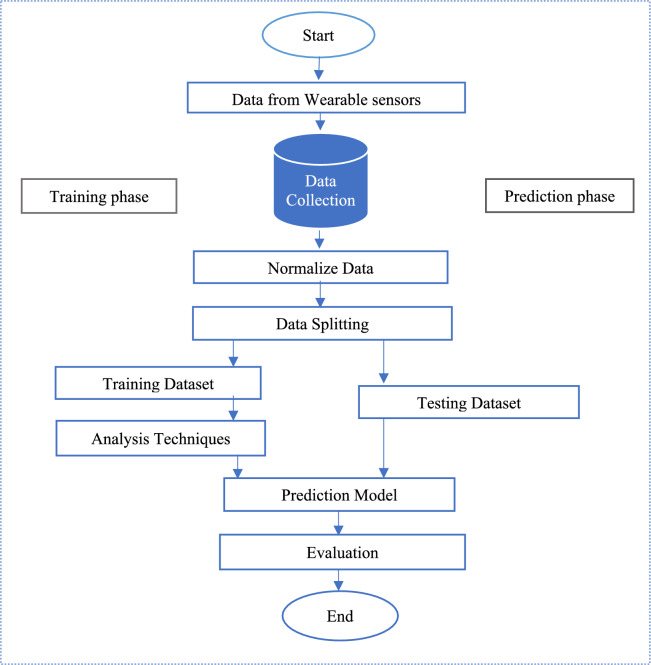
Flow chart of proposed SERS.

The collected dataset is split into training and testing dataset as shown in Fig. 6. In our proposed SERS, the training dataset is labelled by an expert by establishing a link between the internal body parameters to their relevant emotions. The labelled training dataset is used to train the model. After the model training, the trained model is test on the testing dataset and predict the output.

### Data collection

The data acquisition process of the proposed IoT-based smart emotion recognition system aims to acquire multiple internal body parameters using wearable biosensors. These are body temperature, blood pressure, blood glucose, oxygen concentration (SpO₂), muscle signals (EMG), and pulse rate. Figure 5 shows the system architecture.

A multi-sensor wearable system, in the shape of a smart armband, is employed to non-invasively record the physiological signals of the human body. Each sensor is tasked with capturing specific parameters of the body.

**Fig. 7:**
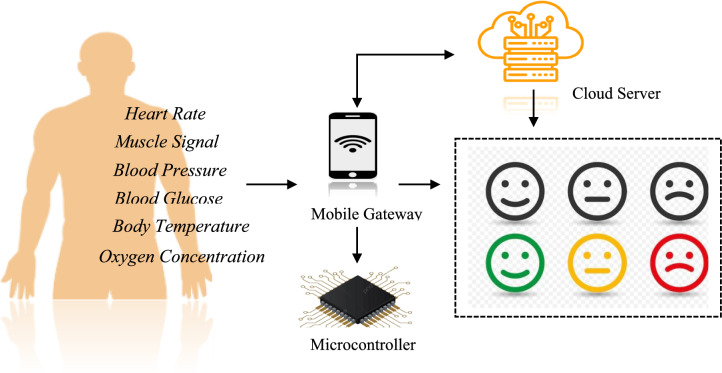
Data acquisition architecture of the proposed SERS.

Figure 7 represents the data acquisition architecture of the proposed SERS. Body Temperature Sensor Tracks variations in skin temperature, which may fluctuate with stress, fear, or excitement. Blood Pressure Sensor Measures systolic and diastolic readings, indicating emotional tension or relaxation. Blood Glucose Sensor Measures glucose variations which might correlate with mood or stress-induced metabolic reactions.Pulse Oximeter Provides oxygen saturation (SpO₂) measures that might decrease in anxiety or affective arousal. EMG Sensor Captures muscle activity from facial or forearm muscles, which respond to affective expressions. Pulse Rate Sensor Measures heartbeats per minute, which are directly influenced by emotional states like happiness, fear, or anger.

All the signals coming from the sensors are channeled through a microcontroller. The preprocessed data is then sent to a mobile gateway (tablet or smartphone) using Bluetooth or Wi-Fi. The mobile gateway takes care of data buffering, temporary storage, and secure transmission to the cloud server.After uploading to the cloud, the physiological data is tagged and warehoused in a central database. It is then employed as input to a machine learning model to classify emotions. Emotions are classified as neutral, happy, sad, fear, anger, and surprise based on pre-defined patterns within the measured internal body parameters.

This sensor-based data acquisition framework allows for real-time, continuous, and non-invasive monitoring, forming the foundation for accurate and scalable emotion recognition across various environments. The model predict the emotion state relevant to the internal body parameter values. In continuous monitoring of real time data, a digital report can be generate by the system that will indicate the variation of emotions with the time interval.

The purposed system is validated by experiment. In the experiment, emotions are induced by using standard dataset then collect the internal body parameter values. The collected values then checked on our proposed SERS system and also validate it by filling a questionnaire from the subject.

#### Ethical approval and informed consent

All procedures were performed according to applicable guidelines and regulations as accredited by the Human Ethics Committee of The Islamia University of Bahawalpur (Approval ID: 241/ORIC). All experiment protocols were approved by the Ethics Committee of the Office of Research, Innovation, and Commercialization (ORIC) at the Islamia University of Bahawalpur. Informed consent was sought from all individual study participants. All data obtained were de-identified to preserve participant confidentiality

### Data storage

In an IoT-based Smart Emotion Recognition System (SERS), secure and reliable storage of data is essential for maintaining the integrity and accessibility of internal body parameters collected from various sensors. The monitored parameters—body temperature, blood pressure, blood sugar level, oxygen saturation, heart rate, and muscle signals—generate continuous streams of sensitive, large-volume data. Therefore, a robust storage system is necessary to support real-time analysis, historical tracking, and secure access. The system employs a hybrid storage structure, making use of local databases for short-term storage as well as cloud storage from Microsoft Azure for long-term storage and high-level analytics.

The sensor data collected is temporarily kept in a local lightweight database like SQLite inside the IoT device or gateway. This temporary storage provides low-latency access, offline processing, and buffering during periods of intermittent connectivity and keeps the system working and responsive in real time.

**Fig. 8:**
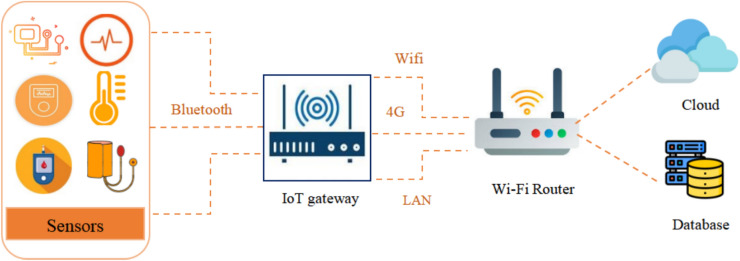
Data storage on cloud and local database.

Figure 8 represent the data storage on cloud and local database. Once data is preprocessed or a reliable connection is established, it is sent securely to the Azure cloud via communication protocols like HTTPS. The data is stored and consumed through Azure Cosmos DB, a managed NoSQL database service for accommodating time-series and semi-structured health data. Cosmos DB provides low-latency access, auto-indexing, and global distribution, critical to real-time emotion detection and big data analytics at scale.

Concurrently, Azure SQL Database is utilized for storing structured elements like system configurations, user profiles, and labeled emotion data sets. This two-tiered storage strategy offers flexibility for handling both dynamic sensor inputs and fixed records with predictable performance.

Furthermore, Azure Backup and Site Recovery services ensure data durability and system resilience by virtue of automated backups, redundancy, and disaster recovery processes. To aid usability of data, every record is stamped with a time, tags, and indexed to facilitate fast querying, monitoring, and machine learning pipelines.

This combined data storage solution not only guarantees the system’s reliability, scalability, and security but also provides a strong foundation for sophisticated data analytics, training deep learning models, and individualized health insights.

### Data preprocessing and data cleaning

#### Data selection

When selecting in the data selection phase, we select only the correct and useful data from the vast information obtained by the sensors. As the system captures numerous readings from body parameters such as temperature, blood sugar, blood pressure, heart rate, and muscle signals, some of that data may be erroneous, out of range, or irrelevant.

To ensure that we utilize only true and meaningful information, we implement medical and logical rules in Table 3.

**Table 3:** Body parameter value range.

Blood sugar (mg/dL)	70 ≤ G ≤ 200
Heart rate (bpm)	35 ≤ HR ≤ 180
Body temperature (°F)	90 ≤ T ≤ 106
Oxygen concentration(%)	75 ≤ OC ≤ 100
Systolic blood pressure (mmHg)	80 ≤ BP_sys_ ≤ 220
Diastolic blood pressure (mmHg)	45 ≤ BP_dia_ ≤ 120
Muscle signal (normalized unit)	0.0 ≤ EMG ≤ 2.00.0

Initial filtering is performed based on predefined physiological thresholds to ensure only relevant and realistic values are retained. The following ranges were used:

#### Cleaning

The data gathered is cleaned to eliminate noise, inconsistencies, and invalid input:

**Missing values:** Interpolation (linear or nearest-neighbor) is utilized to replace short gaps in readings. Multiple missing field records are removed.

**Outlier detection:** Unusual or biologically impossible values (e.g., heart rate > 200 bpm or blood sugar > 500 mg/dL) are detected by Z-score or IQR methods and deleted or replaced by median values.

1. Missing values x_miss_ are replaced using **linear interpolation**
  1 $$xmiss=xi-1+\frac{xi+1 -xi-1}{2}$$
2. If multiple consecutive values are missing, forward-fill is applied:
  2 $$xt=xt-1 if xt is missing$$
3. Outlier detection using Z-score
  For each parameter xxx, the **Z-score** is calculated:
  3 $$Z=x-\frac{\mu }{\sigma }$$

where μ is the mean and σ is the standard deviation of the feature. Values with ∣Z∣ > 3 are treated as outliers and removed or replaced using median imputation.

**Typographical errors:** Manual or computer checks are run to correct errors in entry (e.g., an extra digit in blood pressure or an incorrect decimal in glucose).

**Normalization of units**: All the measurements are brought to standard units (e.g., temperature in °F, glucose in mg/dL, blood pressure in mmHg) to maintain uniformity from data sources.

#### Data normalization

To bring all features onto a uniform scale, Min–Max Normalization is applied:

4 $${x}^{{{\prime}}}=\frac{x- xmin}{xmax-xmin}$$

where, x is the original value, x_min_ is the minimum value, x_max_ is the maximum value, x’ is the normalized value.

#### Data validation

Once normalized, the data is validated to ensure medical and statistical acceptability of all values:

After that, the data is validated for medical and statistical acceptability. This process checks the data for acceptable values. Once the data is normalized, it

Range validation: Each parameter is revalidated to ensure the data is within reasonable and human body limits.

Consistency checks: The data is verified for sudden changes or falls between two consequitive entries, during which there could be anomalies in sensor readings or data recording.Pairs of values are checked, for example a very high heart rate should not be found simultaneously with a very low blood pressure in normal cases. The irregular patterns are marked for screening.

### Data analysis

In the Smart emotion recognition System(SERS) model, after the data preprocessing a dataset has been collected, now the dataset is divided into two separate parts: a training dataset and a testing dataset. This division is essential for developing and validating the machine learning models that will be used to identify emotions based on internal body parameters such as body temperature, blood pressure, blood sugar, oxygen concentration, heart rate, and muscle signals.

**Fig. 9:**
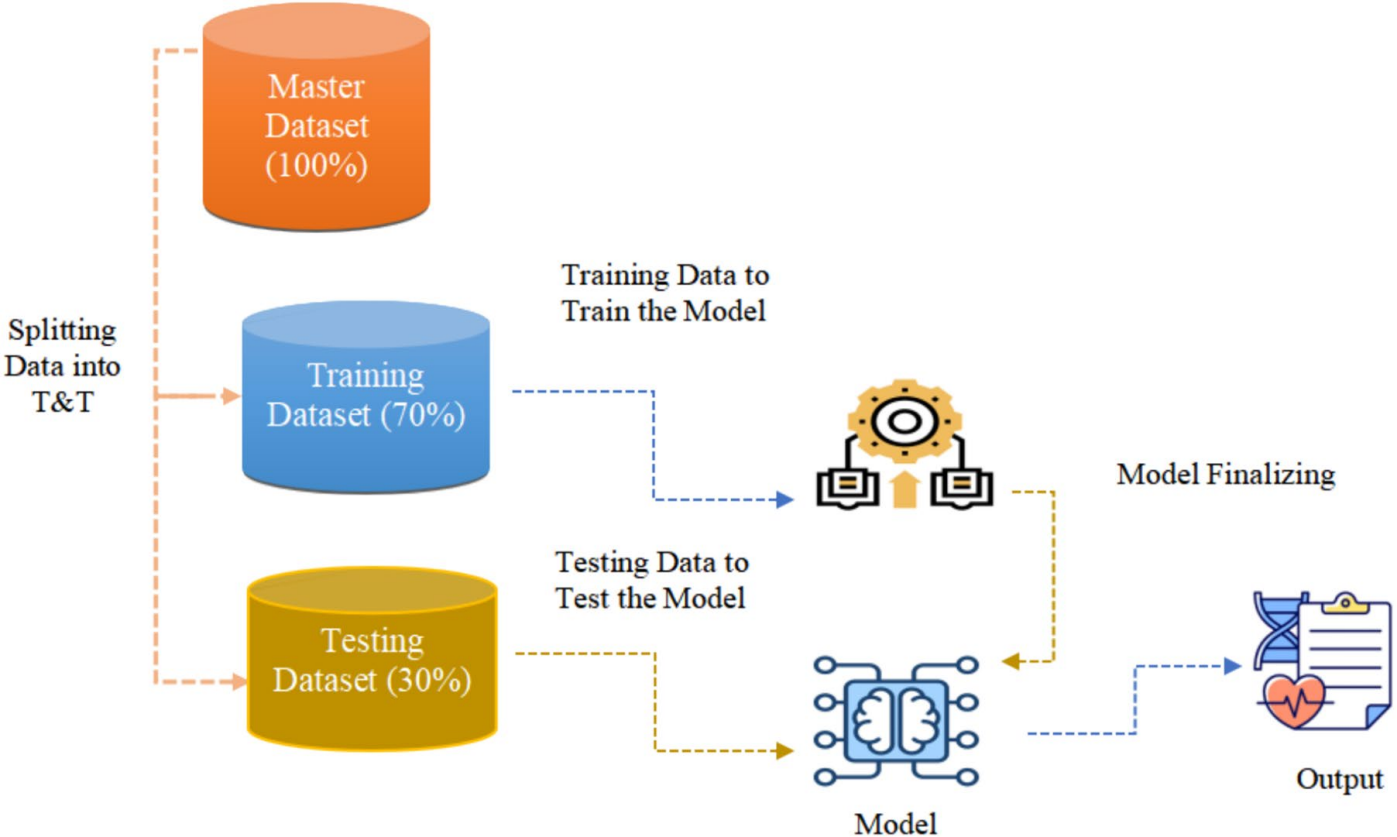
Splitting the dataset into training and testing data.

In Fig. 9 dataset is split into a training and a testing set. In the analysis phase, the preprocessed and cleaned physiological dataset is utilized to train and evaluate a machine learning model for emotion identification. To guarantee correct and equitable assessment, the dataset is split into two halves: 70% for training the model and 30% as the testing set. Splitting prevents overfitting and allows the model to perform well in new, unseen cases. A machine learning algorithm is used to train the model.

Upon training, the model is tested with the held-out testing data. Testing is done by providing the input features (body parameters) of the test data to the trained model, and then having the trained model predict the emotion labels. These predicted labels are compared with the test data actual labels in order to measure the performance of the model.

### Model validation

#### Internal validation

##### Cross validation (K-fold)

To make sure that the proposed Smart Emotion Recognition System (SERS) is robust and has the capability to generalize well, a stratified k-fold cross-validation technique is used in this research, and the k is set to 5. While performing stratified k-fold cross-validation, all folds are ensured to have more or less equal numbers for the six classes (neutral, happy, sad, fear, anger, and surprise) defined for this research.

In this method, a partitioning of the dataset into five non-overlapping folds was carried out. In every run, four folds are used for training, while the other fold is used for testing. This is repeated five times, with every fold being used exactly once for testing. Finally, the performance of the model was calculated based on averages of all folds, allowing for a concrete estimate of performance and consistency of models.

From the experimental evaluation of various machine learning classifiers, a Random Forest classifier was chosen for cross-validation and further validation based on its capacity for ensemble learning and its ability to handle nonlinear relationships and variability present within physiological signals. For cross-validation, performance of the model was measured by use of common assessment metrics such as accuracy and weighted F1 score.

#### External validation

##### Model validation using DEAP stimuli

To test the generalization ability of the proposed Smart Emotion Recognition System model on the new dataset other than the dataset collected from within the organization, an external model validation test was conducted with the DEAP dataset. A total of 17 participants voluntarily participated in the external model validation test.

The emotional induction procedure utilized the selected videos and pictures of the DEAP database, which are commonly employed to provoke and study the participants’ emotional activation in a controlled manner. The internal physiological conditions of the participant’s body, with the emphasis on the level of the body’s temperature, blood pressure, blood sugar condition, level of blood oxygenation, heart rate, and muscular activity, were then measured with the same sensor configuration as in the main data collection procedure.

The physiological signals obtained were then preprocessed, and the feature extraction step was carried out through the same process used in modeling. A Random Forest classifier trained on internal validation sets was then directly used on the newly obtained dataset without the need for retraining.For the purpose of examining the efficacy of the proposed framework on the externally induced emotion data, standard classification measures such as accuracy, precision, recall, and F1-score values are used.

## Implementation

### Hardware components

A system employing IoT utilizes embedded sensing devices for the efficient and cost-effective sensing and recording of real-time data^37^. We also used sensors for real time data sensing. Our proposed SERS, used sensors based circuit for real time monitoring of internal body parameters which then provided to machine learning model for prediction after preprocessing. The hardware components used in data collection circuit are depicted below:

Arduino UNO Microcontroller

- Ds1820 Body Temperature Sensor
- Max30100 Blood Oxygen Concentration Sensor
- Heart Rate Sensor
- Electromyography Sensor
- Blood pressure Sensor
- Blood glucose

**Fig. 10:**
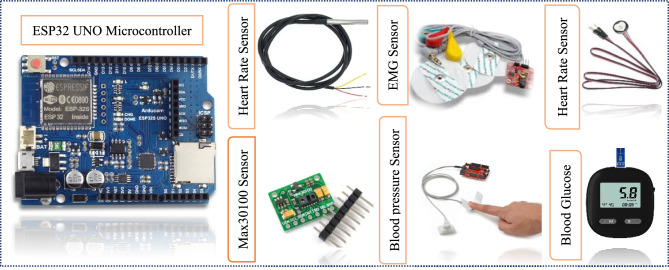
Hardware components in SERS.

Figure 10 represent the hardware components in SERS. Our designed circuit used above hardware components. We used six sensors Ds1820 sensor for measuring the body temperature, Max30100 sensor for measurement of blood oxygen concentration, pulse sensor for measuring the heart rate variability for collecting internal body values, EMG sensor for measurement of electric signal of muscle, Galvanic skin Response for measurement of skin Conductance and Blood glucose sensor for measurement of blood glucose level.

#### ESP UNO microcontroller

The UNO ESP32 is the central processing unit of the proposed IoT-based Smart Emotion Recognition System. This is a dual-core 32-bit microcontroller having on-board Wi-Fi and Bluetooth connectivity, ensuring wireless real-time data transmission to the Microsoft Azure cloud platform. The ESP32 collects data from the following physiological sensors: BP, temperature, SpO₂, heart rate, and EMG sensors, through the analog and digital pins, respectively.

It offers high processing speed with low power consumption and is cost-effective, hence very suitable for continuous monitoring. An integrated 12-bit ADC provides precise signal acquisition while its deep-sleep mode enhances battery performance.

Advantages: fast processing, wireless connectivity, energy efficiency, and scalability for multi-sensor integration. Limitations: It is sensitive to power fluctuations; it requires precise configuration so that data transmission is stable.

#### Blood pressure (BP) sensor

The blood pressure sensor measures systolic and diastolic pressure either by detecting arterial oscillations during cuff deflation or through piezoresistive pressure transducers. For this work, the sensor provides continuous non-invasive readings interfaced to the ESP32 through an analog input pin.

**Advantages:** It provides reliable information about cardiovascular activity, as changes in BP are strongly associated with the stress, anger, and fear responses.

**Disadvantages**: Susceptible to motion artifacts and calibration drift; continuous BP monitoring may consume more power.

#### Electromyography sensor

The EMG sensor measures muscle activation signals by detecting the electrical potential that is generated during muscle contractions. Emotional states, such as anger or stress, are generally associated with increased muscular tension, especially in facial or forearm muscles.

**Advantages:** Direct insight into muscle response and tension; sensitive to subtle emotional changes.

**Disadvantages**: Requires skin preparation and careful electrode placement; may capture noise from surrounding muscles and electrical interference.

#### Body temperature sensor

The body temperature sensor (DS18B20) measures peripheral temperature changes with thermoelectric sensing mechanisms. Emotional states such as stress, fear, or excitement will induce slight but detectable temperature changes due to changed blood distribution.

**Advantages**: Small, inexpensive, and low power consumption; offers linear and stable temperature response.

**Disadvantages**: Inclined towards ambient temperature; necessitates good skin contact for reliable readings and can exhibit latency in responding to quick emotional shifts.

#### Blood sugar sensor

This blood sugar sensor gives a measure of the glucose concentration level in the blood, which may change with changes in emotional or physiological states such as stress or anxiety. The sensor basically uses the principles of enzymatic electrochemical reactions, where GOx reacts with glucose to provide an electrical response proportional to the glucose level. The sensor interface in this system connects to the analog input of the ESP32 for continuous data monitoring.

**Advantages:** Provides accurate quantative data, suitable for real time health and emotion analysis.

**Disadvantages:** Needs periodic recalibration; temperature, humidity, and sensor degradation with time may eventually affect accuracy.

#### Pulse rate sensor

The heart rate sensor, often integrated with the SpO₂ module, detects pulse rate using PPG signals. Emotional variations such as excitement, anger, or fear often cause rapid fluctuations in heart rate.

**Advantages**: Simple, inexpensive, and reliable in short-term monitoring; can be easily worn for continuous data collection.

**Disadvantages** include that motion noise and improper sensor placement may distort the readings; signal filtering and preprocessing are required to reduce the artifacts.

### Data sensing

**Fig. 11:**
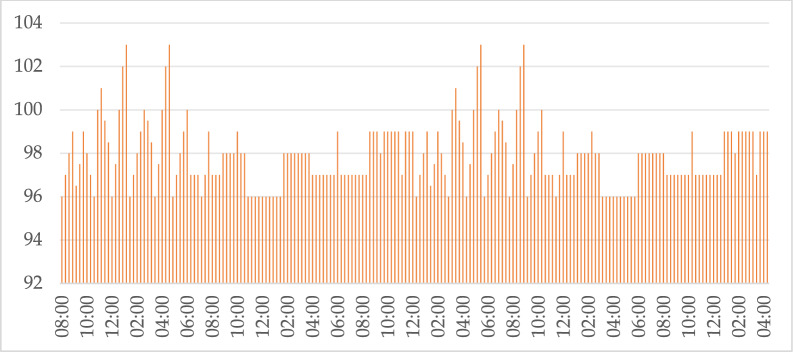
Temperature values in SERS.

The first step of the proposed system is sensing data from sensors. In the Issued-IoT-based methodology framework, sensing is considered the first and most primary level for raw data acquisition from the real physical world. In the context of healthcare systems and/or emotion recognition, this level comprises a set of biomedical sensors implanted on a human body for sensing internal human physiological parameters like heart rate, human body temperature, human blood pressure, oxygen saturation, human glucose levels, and human muscle signals as Fig. 11 showing the values of temperature of different participants.

These sensors track the body’s physiological signals in real time and generate immediate data, which is used as input for the higher layers of the IoT system. The sensing layer serves as the interface between the physical body and the digital system, enabling the acquisition of vital information for further analysis.

This aggregated data is then transmitted to the network layer for communication and subsequently to the processing and application layers for analysis, visualization, and decision-making. The success of the entire IoT architecture relies heavily on the responsiveness and accuracy of the sensing layer, making it a crucial component of any health or emotion recognition system.

**Fig. 12:**
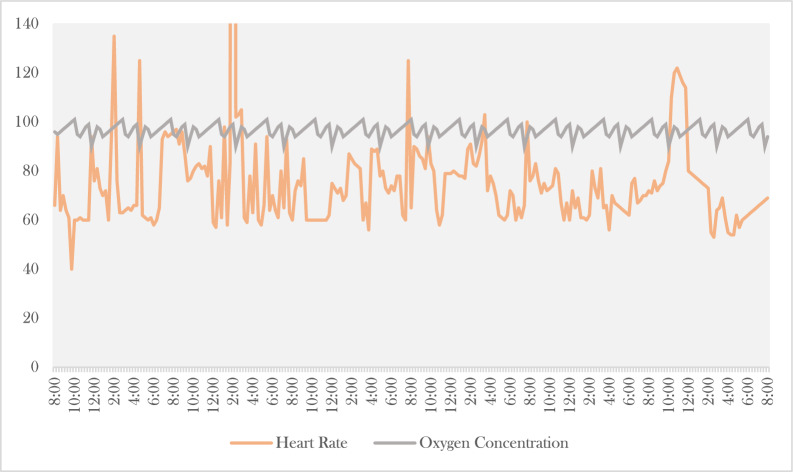
Heart rate and oxygen concentration values in SERS.

**Fig. 13:**
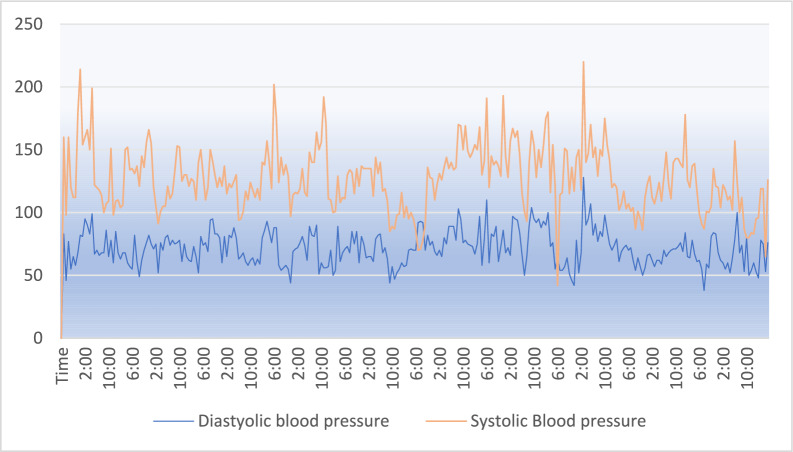
Diastolic blood pressure and systolic blood pressure.

**Fig. 14:**
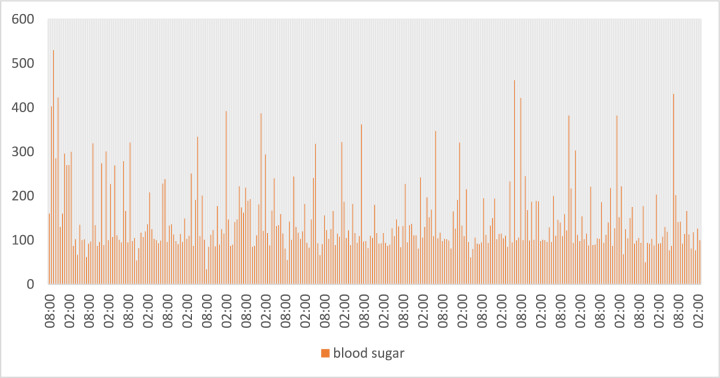
Blood Sugar values in SERS.

**Fig. 15:**
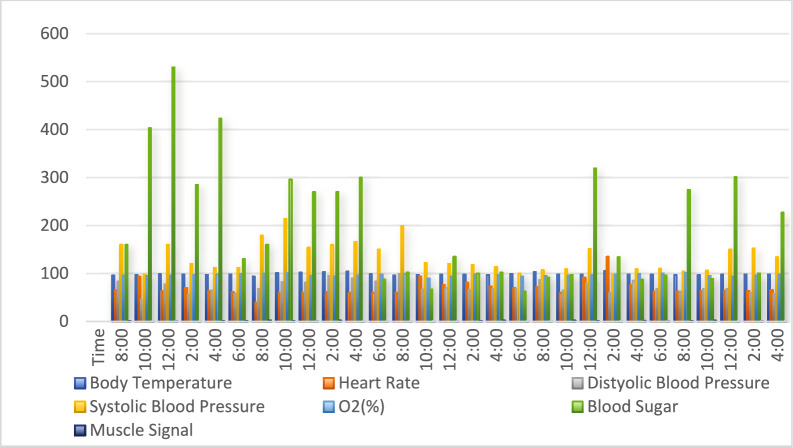
Data collection of different body parameters by using IoT framework.

Figure 11,12,13,14 and 15 indicate how real-time data is collected in the SERS system using IoT framework. The data of different body parameters is collected and integrated on one place for further acttions.

**Fig. 16:**
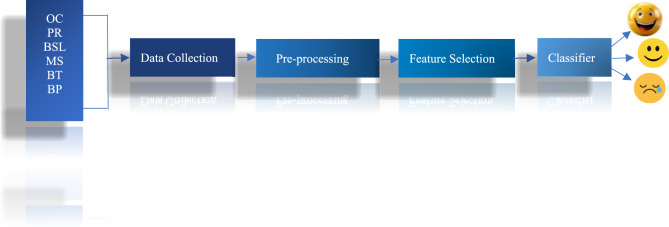
Diagram of data processing stages of emotion Recognition.

In this study, emotions were not artificially evoked with pre-defined stimuli. Rather, physiological measurements were made in natural, real-time environments so that emotions occurred spontaneously during participants’ own daily activities. This way, genuine fluctuations in internal body measures that correspond to naturally arisen emotional states were captured.

To guarantee reliability and accuracy for labeling, every segment of data was labeled by domain experts and psychological consultants according to contextual clues, participant reports, and physiological signal patterns. The emotion labels obtained (neutral, happy, sad, fear, anger, and surprise) were cross-validated against the DEAP dataset for identification with existing physiological-emotional patterns and uniformity with known standards.This technique offered a realistic but guided labeling process, reducing the biases that can occur due to artificial emotion induction techniques.

### Data set

To conduct data acquisition, experiment was performed in a closed and soundless room. To enhance data acquisition, experiment was performed under similar environment conditions for each subject. In a bid to get a quality dataset of physiological signals, a specific setup was developed for conducting an experiment, including setting up instruments for conducting an experiment and choosing subjects for an experiment. In regard to subjects’ privacy, sought their consent prior to data acquisition. This aspect acts as a crucial dimension, indicating how subjects’ data was dealt with during an experiment.The distribution of instances in the dataset is illustrated in Table 4.

**Table 4:** Dataset features.

Sr.#	Attribute	Datatype	Description	Values
1	Participant-ID	Integer	Unique id for each participant	1,2,3,….etc
2	Name	Object	Name of Participant	ABC,XYZ..etc
3	AGE	Integer	Participant Age	22,24,26,…etc
4	TEMP	Integer	Temperature in Fahrenheit	96,97,98…..etc
5	BSR	Integer	Blood Glucose Level(Sugar)	119,165,140…etc
6	PULSE	Integer	Pulse Rate	65,75,80..etc
7	BP	Integer	Systolic/Diastolic blood pressure	80/120,85/130..etc
8	O2(%)	Integer	Blood oxygen level in	96,97,98…etc
9	EMG	Integer	Muscle signal	0,1,2

The dataset contains a total of 10 attributes, including general and sensor-related ones, and 3600 instances in total. A detailed description of the dataset features is given in Table 4. 7 features are integer type and three features are object type.

### Dataset preparation

A preprocessing step is applied on the dataset and labeled according to certain rules that have been designed prior to the training process of the RF classifier. These rules are designed with the observation of medical experts. The attributes belonging to the dataset of the patient information, namely the Patient _ ID, NAME, and AGE attributes, are removed as they do not contribute towards the ability of the RF model in result predictions. All the data is collected from the participants of the aged group belonging between 20–80 years. This process involves the data collection process of the 4000 participants. For the sensor-related attributes of the dataset, the data is labeled on the basis of individual rules designed separately for the sensor-related attributes. This dataset with sensor-related attributes and some of the data unlabeled is shown in the Table 5.

**Table 5:** Dataset.

Gender	Age	Body temperature (F)	Blood pressure (mmgh)	Pulse rate (BPM)	Oxygen concentration (%)	Blood glucose (mg/di)	Muscle Signal
Male	22	95	75/122	65	90	120	0
Female	45	97	80/130	70	91	130	1
Male	39	96	90/125	72	95	144	1
Male	55	98	95/132	75	96	220	2
Female	51	99	77/129	76	99	150	0
Male	55	100	85/139	70	98	170	1
Male	65	101	92/141	79	97	160	0
Male	34	96	79/130	80	93	167	1
Female	47	102	88/138	67	97	270	0
Male	23	103	100/150	81	99	299	1
Female	29	98	71/110	77	89	199	1

Concerning the pulse sensor data, the classification process is accomplished by the utilization of rules as described in Table 6. Four classes (Normal, High, Threaten, Dangerous) are assigned to the respective value range of the pulse rate data measured by the Pulse rate sensor.

**Table 6:** Pulse rate sensor data labeling based on value range.

Pulse rate ( BPM)	Class
Pulse < 50BPM	Very low
Pulse < 65 BPM and > = 50 BPM	Low
Pulse < 80 BPM and > = 65 BPM	Normal
Pulse > 80and < = 98 BPM	High
Pulse > 98BPM	Very high

For the body temperature sensor data, the categorization of data is performed using the rules shown in Table 7. Four classes (Normal, High, Threatening, and Dangerous) are assigned to the corresponding value range of the body temperature data from the temperature sensor.

**Table 7:** Temperature sensor data labeling based on value range.

Temperature ( F)	Class
Temperature < 90F	Very low
Temperature < 93F and > = 90 F	Low
Temperature > 95 F and < = 99 F	Normal
Temperature > 99 F and < = 103 F	High
Temperature > 103 F	Very high

For the oxygen concentration data, the labeling follows the rules described in Table 8. There are five classes (Neutral, Low, very Low, High, very high) assigned to the corresponding value range of the oxygen concentration data measured from the Max30100 sensor.

**Table 8:** Max30100 sensor data (oxygen concentration) labeling based on value.

Oxygen concentration (%)	Class
O2 < 92%	Very low
O2 < 95% and O2 > = 92%	Low
O2 > 95% and < = 99%	Normal
O2 > 99% and < = 102%	High
O2 > 102%	Very high

For data from a blood glucose sensor, the categorization of labels is performed based on rules described as follows: Table 9. There are four classes of labels: Normal, Low, High, and very High(Dangerous). These labels are marked based on the value range of the data from a blood glucose sensor.The dataset is preprocessed and labeled based on certain criteria that have been developed under For the data of the blood pressure sensor, the labeling is performed using the rules as illustrated in Table 10. Four categories (Normal, Low, High (Stage 1 Hypertension), and Very High (Stage 2 Hypertension) are mapped into the corresponding ranges of the systolic and diastolic blood pressure data measured by the blood pressure sensor.

**Table 9:** Glucometer sensor data (blood glucose) labeling based on value range.

Blood Glucose (mg/dl)	Class
BSR < 60 mg/dl	Very low
BSR < 80 mg/dl and > = 60 mg/dl	Low
BSR > 80 mg/dl and < = 150 mg/dl	Normal
BSR > 150 mg/dl and < = 180	High
BSR > 180 mg/dl	Very high

**Table 10:** Classes based on the blood pressure(systolic and diastolic) range value.

Systolic(mmgh)	Diastolic(mmgh)	Class
BP < 100mmgh BP < 115mmgh and > = 100mmgh	BP < 55mmgh BP < 70mmgh and > = 55mmgh	Very Low Low
BP > 115mmgh and < = 130mmgh	BP > = 75mmgh and < = 85mmgh	Normal
BP > 130mmgh and < = 140mmgh BP > 140mmgh	BP > 85mmgh and < = 95 BP > 95mmgh	High Very high

For Muscle signal data, the labelling is done using the rule in Table 11^38^. Three classes (Normal, Stretched and very stretched ) and assigned to the corresponding value range.

**Table 11:** Classes based on the muscle signal range value.

Muscle signal (V)	Class
Muscle signal amplitude < 1 V	Relaxed (normal)
Muscle signal amplitude > 1 V and < 2 V	Stretched (high)
Muscle signal amplitude > 2 V	Very stretched (very high)

**Table 12:**
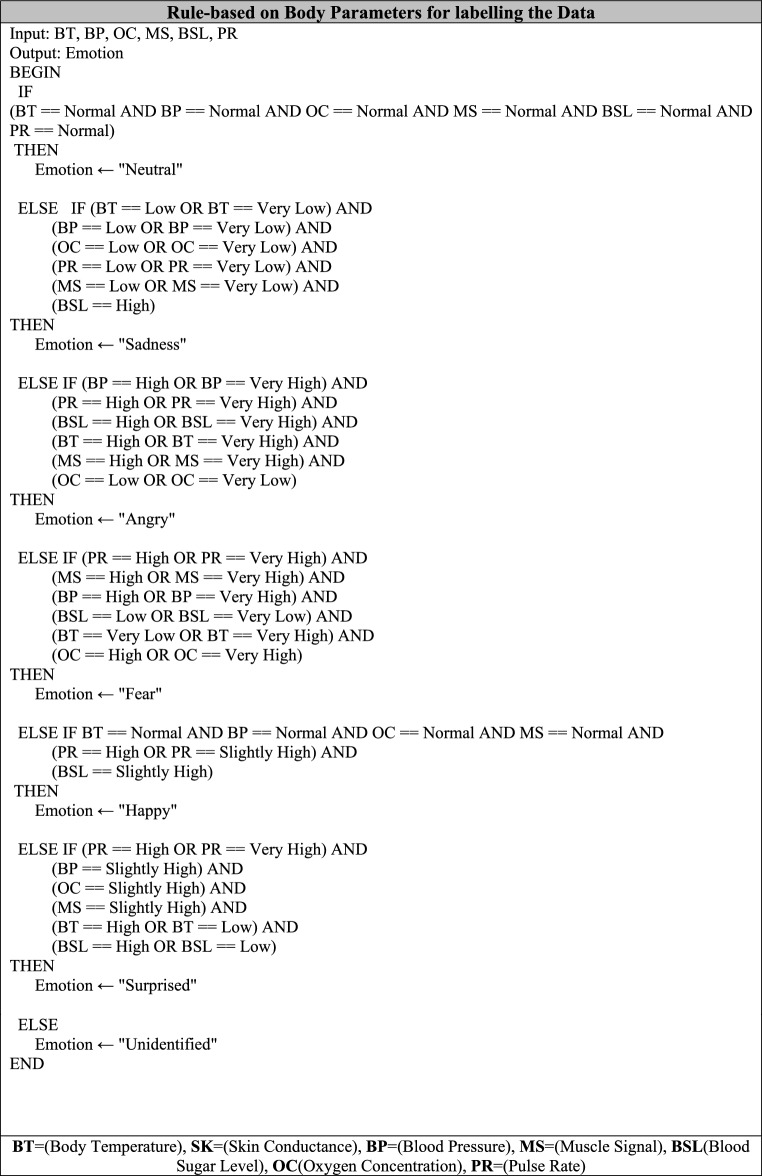
(Pseudo) Defined rules for creating output label attribute (condition).

The dataset labelling done with the help of expert/consultant. The real time dataset is collected of 4000 participants and then then labelled according to above rules mention the Table 12.

### IoT deployment and system scalability

The suggested IoT-based Smart Emotion Recognition System uses ESP32 microcontrollers for real-time acquisition and wireless communication of physiological parameters, such as heart rate, temperature, blood pressure, oxygen level, and muscle activity, to Microsoft Azure cloud for processing and storage.

The ESP32 board was selected due to its low-power, low-cost, and Wi-Fi-capable architecture that allows for easy interconnectivity with the Azure IoT platform. The average end-to-end latency from data collection to cloud update was noted to be less than 200 ms while undergoing experimental evaluation, proving appropriateness for near real-time healthcare monitoring.

In order to provide communication reliability, the system incorporates edge device local buffering and periodic cloud synchronization to limit packet loss during momentary disconnections. In energy efficiency, the ESP32 runs in low-power modes during idle times to prolong device operation for tens of hours on a typical Li-ion battery.

For scalability purposes, the Azure cloud platform accommodates the integration of numerous IoT nodes, where data handling is facilitated simultaneously for multiple users. This organization allows the proposed framework to scale well for ongoing, real-world healthcare monitoring scenarios like hospital settings or remote patient monitoring.

### Used analysis techniques

In our proposed SERS, Internal body parameters are read by an Arduino-based circuit used for analysis, to predict emotions. Numerous machine learning techniques are accessible for data analysis to forecast outcomes, including Random Forest, SVM, Gradient boosting, and KNN. Classification algorithms find diverse applications in addressing healthcare issues e.g. stress management systems, safety systems^39,40^.

n our proposed system, we used different Machine Learning classifiers e.g. SVM(Support Vector Machine), RF(Random Forest), AdaBoost(Adaptive Boost) and KNN(K-Nearest Neighbor). A description of these algorithms is given below.

(1) **Random forest**

The Random Forest operates by utilizing an ensemble of decision trees within a supervised learning framework. In terms of speed, it outperforms other supervised learning techniques. The outcomes of individual trees are assessed through either majority voting or average calculation. This algorithm demonstrates a commendable performance metric. Typically, Random Forest (RF) serves as a probabilistic predictor in classification tasks, which are characterized by:

5 $$Y\left(x\right)= \frac{1}{NT }\sum_{i=0}^{NT}{R}_{i}\left(x\right),$$

where x represents the vector of the input, and Ri(x) be the regression tree of *i*.*NT* defines the trees number. Second, the random forest model is less likely to overfit since the extra-trees (ET) model is a developed variation of the random forest. To help the estimators perform better, they can choose at random the best characteristics from the input dataset’s^41^.

Among a number of machine learning methodologies that were employed in this study, Random Forest Classification is one of the most powerful methods for predictive analytics. The method trains within the category of ensemble learning, which is renowned for its capacity for improving prediction precision and handling complicated data sets, often encountered in industrial environments^42^.

Forming an ensemble, which is also referred to as a high number of decision trees, is the first step in the Random Forest Classification procedure. Every decision tree is built from a different subset of the original dataset, and it works independently and makes its own set of predictions. The bagging process, otherwise known as bootstrap aggregation, provides each tree a chance to “vote” for the ultimate forecast^43^.

One of the most significant features of Random Forest is that it adds randomness to the decision tree development process. Both the fact that we select a random subset of features for each tree and the fact that we use a different subset of the training data for every tree are the main contributors to this randomness. To begin, we use a different subset of the training data for each tree. Due to the reason that each decision tree is unlike the rest, the overall classification becomes stronger and more precise due to this diversity^44^.

**Fig. 17:**
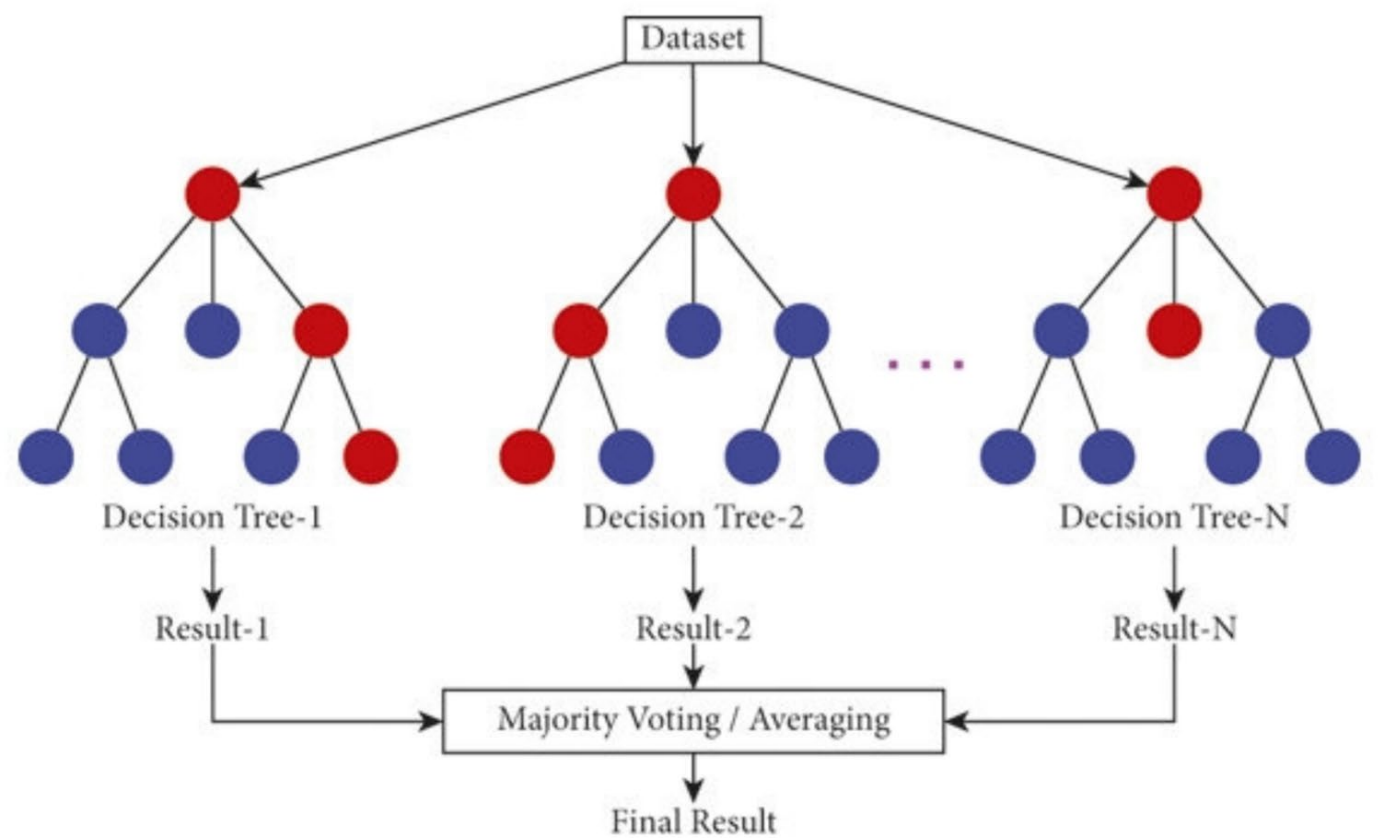
Random forest classifier.

Figure 17 shows the model of Random Forest. Due to its resistance to overfitting, flexibility with high-dimensional data, and ability to handle complex relationships in the dataset, Random Forest Classification is a great option for solving problems that occur in industrial IoT systems. We believe that with its inclusion in our research, we will be able to enhance the predictive abilities of industrial IoT systems and make them more intelligent^45^.

K- Total number of features that are in the data-set.

X- the feature matrix.

Y- the target variable (class labels in classification).

N-the number of decision trees.

In Random Forest, each decision tree is given a randomly chosen subset of features, which brings in the element of randomization. The following is one way we can describe it, where m = K is the size of the random subset. Bootstrapping is a method where decision trees are trained on a forest setting^46^. The process involves randomly choosing a subset of the original dataset and then replacing instances in the subset. Below is an illustration of the process, which explains the probability of being selected as a data point in the bootstrap sample as 1/N:

6 $$P\left(Selected\right)=\frac{1}{N}$$

In the training of every decision tree i, it uses the bootstrap sample. At every decision tree node, the data is divided according to the Gini impurity or the entropy.

7 $${\mathrm{G}}{\mathrm{.I}}{\mathrm{.}}\left( {\mathrm{f}} \right) = 1 - \sum {_{{\mathrm{i}}} = 1{\mathrm{k}}} _{{{\mathrm{(p}}_{{\mathrm{i}}} )^{2} }}$$

where, K- is the number of classes. Pi- proportion of instances of class i in the node. In the classification step, every decision tree votes for a particular class; the final prediction is the class that receives the most votes from all trees.

8 $${Y}{\prime}=argmaxc\sum i=1NI\left({Y}{\prime}i=k\right)$$

where, Y′i —prediction of the i-th tree. k- class label. I-indicator function.

**Algorithm 1:**
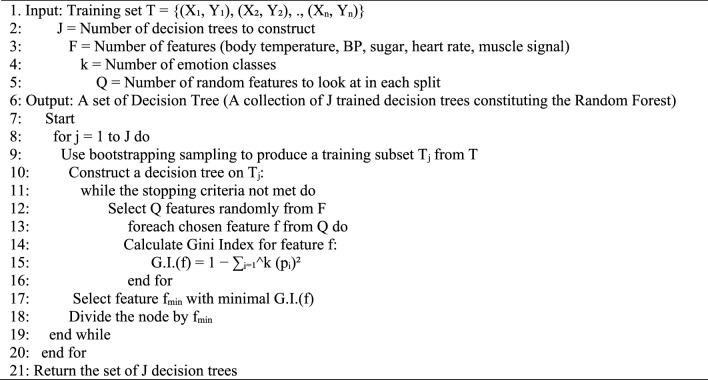
Build Random Forest (T, F, J)

(2) **K-Nearest Neighbor**

The K-nearest neighbor (KNN) algorithm is a classification method rooted in comparative learning. When applied to experimental data, the algorithm identifies k samples from the rfmnearest neighbors. This method determines the new class to which an instance belongs by assigning it to the class that receives the most votes from its K nearest neighbors^7^. The K-nearest neighbor (KNN) method is a simple statistics-based classification method, which is commonly used in mature classification algorithms^47^.

The similarity between data points measured using the **Euclidean distance**:

$$d\left({x}_{i},{x}_{j}\right)=\sqrt{\sum_{k=1}^{n}{\left({x}_{ik}-{x}_{jk}\right)}^{2}}$$

where ${x}_{i}$ and ${x}_{j}$ are feature representing physiological samples.

n is the number of features.

The predicted class $\widehat{y}$ for a new instance $x$ is determined by the **majority voting rule**:

$$\widehat{y}={\mathrm{mode}}\{{y}_{i}\hspace{0.17em}|\hspace{0.17em}{x}_{i}\in {N}_{k}\left(x\right)\}$$

where ${N}_{k}\left(x\right)$ represent the set of *k* nearest neighbors of $x$.

(3) **Support vector machine**

SVMs demonstrate effectiveness in both classification and regression tasks, rendering them suitable for applications such as emotion recognition. They particularly excel in scenarios where distinct boundaries exist between various emotional states^48^.

Support Vector Machine (SVM) was utilized in the current research to categorize human emotions from internal body parameters like heart rate, body temperature, blood pressure, oxygen level, and muscle activity. SVM is a supervised learning algorithm that builds an optimal hyperplane to classifies data points from diverse emotional states with the highest margin^48^.

For training data set (xi, yi), where xi represent the physiological features and yi represents the classes of emotions. *w*^*T*^*x* + *b* = 0 ***

And optimized as

$$\underset{w,b}{\mathrm{min}}\frac{1}{2}\parallel w{\parallel }^{2} \text{subject to} {y}_{i}\left({w}^{T}{x}_{i}+b\right)\ge 1$$

For non linear emotion pattern, regularization parameter C were introduced.

$$\underset{w,b,\xi }{\mathrm{min}}\frac{1}{2}\parallel w{\parallel }^{2}+C\sum_{i=1}^{n}{\xi }_{i}$$

To handle complex relationship between physiological signals and emotions, Radial basis functions are used (RBF).

$$K\left({x}_{i},{x}_{j}\right)=\mathrm{exp}\left(-\gamma \parallel {x}_{i}-{x}_{j}{\parallel }^{2}\right)$$

(4) **Logistic regression**

Logistic Regression is a supervised learning machine algorithm applied mainly to binary and multi-class classification tasks. Although it has the word “regression” in its name, it is actually a classification algorithm and not an algorithm for regression. It estimates the probability of a given input belonging to a particular class through a sigmoid function^49^.

In the emotion recognition context, logistic regression is used to predict the likelihood that a series of physiological readings (e.g., heart rate, blood pressure, blood sugar level, temperature, and muscle signals) relate to a particular emotional state (e.g., Happy, Sad, Angry, etc.). The model weights the features and calculates the output as:

$$P\left( {y = 1\left| x \right.} \right) = \frac{1}{1} + e - \left( {\beta 0 + \beta 1x1 + \beta 2x2 + \cdots + \beta nxn} \right)$$

where,

- P(y = 1∣x) is the probability of the positive class
-  × 1​, × 2​,…,xn​ are the input features
- β0,β1,…,βn​ are the model parameters

(5) **Gradient boosting**

Gradient Boosting is a strong ensemble learning method that constructs a robust classifier by aggregating many weak learners (usually shallow decision trees). It iteratively reduces a loss function with gradient descent.

With each iteration, a new decision tree is learned to adjust the mistakes (residuals) made by the existing model. This renders Gradient Boosting very precise and able to learn non-linear relationships, which are prevalent in emotion recognition from internal body parameters.

The model is built in stages:

$$Fm\left(x\right)=Fm-1\left(x\right)+hm\left(x\right)$$

where,

F_m_(x) : Current Model

Hm(x) : the new tree (learner) fitted on the residuals of previous predictions.

(G) Model training

**Fig. 18:**
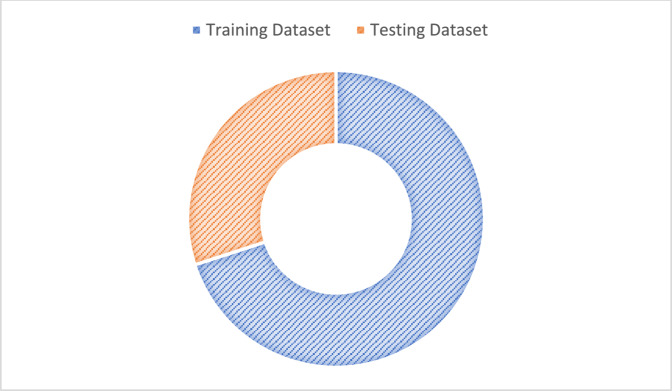
Dataset split: distribution of training and testing datasets.

To train the model with different classifiers, the processed dataset is divided into the training set (75%) and testing set (25%) using the stratified train-test split method. The distribution of the training and testing dataset is shown in Fig. 18.

(H) Hyperparameter optimization strategy

To make a fair and unbiased comparison between classifiers, hyperparameter tuning was performed with grid search with fivefold cross-validation for Random Forest (RF), Support Vector Machine (SVM), and Gradient Boosting classifiers. The grid search approach systematically searched through combinations of pre-selected parameter values, choosing those that resulted in the maximum mean accuracy on validation folds.

For the Random Forest, parameters such as the number of estimators (n_estimators), maximum tree depth (max_depth), and minimum samples split (min_samples_split) were tuned.

For the **SVM**, the kernel type (linear, polynomial, RBF), regularization parameter (C), and kernel coefficient (γ)(\gamma)(γ) were optimized.

For Gradient Boosting, the number of estimators, learning rate, and maximum depth of individual learners were systematically varied.

All models were trained using identical training-test splits to maintain experimental consistency. The optimal hyperparameters obtained through grid search were then used to report final classification results.

## Performance evaluation

For accessing the performance of the classifiers in predicting human emotion, the dataset was collected from different sensors and used for training and testing. The details of the dataset and its preparation, experimental setup for data analysis, training process, and performance metrics are discussed in this section.

### Performance metrics

Several approaches can be employed to assess performance, with techniques such as statistics and mathematics falling within this classification. The designed system’s efficacy, performance, and dependability are assessed through analytical analyses, including the confusion matrix, accuracy, F1-score, precision, recall, sensitivity, specificity, positive predictive value (PPV), and negative predictive value (NPV). The explanation of these performance metrics is outlined below:

(1) **Confusion matrix**

The effectiveness of a classifier on a set of samples with known true values is denoted by a confusion matrix. In the results, a multi-class confusion matrix is detailed for a classification problem involving more than three classes^50^.

(2) **Accuracy**

Accuracy is the capability of the classifier to precisely categorize inputs into their respective categories.

9 $$Accuracy= \frac{TP+TN}{TP+TN+FP+FN}$$

(3) **Precision**

Precision is used to measure how many positive predictions are actually predicted correctly by the classifier. For example, precision can be measured by dividing the total number of positive samples that are correctly predicted (TP) by the total number of samples predicted as positive by the classifier (TP+FP) as shown in:

10 $$Precision= \frac{TP}{TP+FP}$$

(4) **Recall**

Recall is the classifier’s ability to identify every positive sample among all the positive and negative samples^50^. Recall can be measured by dividing the total number of positive samples that are correctly predicted (TP) by the total number of positive samples that are correctly predicted as positive and wrongly predicted as negative by the classifier (TP + FN) as shown in:

11 $$Recall= \frac{TP}{TP+FN}$$

(5) **F1-Score**

The F1 score serves as a crucial metric in evaluating classifiers based on unbalanced datasets, particularly when certain classes have fewer samples^2^. This metric computes the weighted harmonic mean of precision and recall and can be determined using the following formula:

12 $$F-measure= \frac{2*\left(Precision*Recall\right)}{Precision+Recall}$$

(6) **Precision-recall curve**

The precision-recall curve is a highly helpful metric for evaluating the performance of the model, particularly in situations when the classes are unbalanced. The classifier Performance is plotted on a PR curve, which displays precision on the y-axis and recall on the x-axis. The PR curve illustrates the trade-off between precision and recall at multiple points, each of which is evaluated at a different threshold. As the classifier performs better when the average Precision (AP) is greater, the “ideal” position on the plot is in the top right corner with high precision and recall.

(7) **Receiver operating characteristic curve**

A visual representation known as a Receiver Operating Characteristic (ROC) curve, commonly referred to as a simple ROC curve, illustrates the overall performance of a classifier across various potential thresholds. The ROC curve typically plots the True Positive Rate (TPR) against the False Positive Rate (FPR), with the TPR out of the positives on the Y-axis and the FPR out of the negatives on the X-axis. The optimal position on the plot is in the upper-left corner, with an FPR of 0 and a TPR of 1, indicating optimal classifier performance, especially when the Area Under the Curve (AUC) is higher. The TPR and FPR can be calculated using the following equations:

$$TPR= \frac{True Positive Samples Count }{Total Number of Positive Samples} \left(13\right)$$

$$FPR= \frac{False Positive Samples Count }{Total Number of Negative Samples} \left(14\right)$$

(8) **Matthews correlation coefficient**

One of the most useful metrics, known as the MCC, can be utilized to calculate the classifier’s effectiveness for binary or multiclass classification. The MCC is considered a balanced measure because it performs well in those cases where the classes are of different sizes, i.e., imbalanced datasets. The value of MCC for the multiclass scenario ranges between − 1 and 0 as the minimum range limit and + 1 as the maximum range limit, where + 1 indicates the perfect prediction and − 1 as an inverse prediction. The MCC for the multiclass can be computed using:

$${MCC}_{multiclass}=\frac{{{c}_{samples}*n}_{samples}-{\sum }_{i}^{I}{p}_{i}*{t}_{i}}{\sqrt{\left({n}_{samples}^{2}-{\sum }_{i}^{I}{p}_{i}^{2}\right)*\left({n}_{samples}^{2}-{\sum }_{i}^{I}{t}_{i}^{2}\right)}} \left(15\right)$$

where, ${n}_{samples}$ indicates overall samples count, ${c}_{samples}$ indicates corrected predicted sample count, ${p}_{i}$ indicates the number of times class *i* was predicted, and ${t}_{i}$ indicates the number of times class *i* actually happened.

## Results and discussion

A smart and Intelligent system design characterizes the Smart Emotion Recognition system. Different machine learning analysis techniques were used to evaluate the effectiveness of the proposed IoT-based Smart Emotion Recognition System(SERS). The dataset consists of labeled segments corresponding to six emotional states: Happy, sadness, fear, anger, Neutral, and surprise. The data is split into 70% training and 20% testing sets and model performance is evaluated using various performance metrics and visualization techniques.

**Fig. 19:**
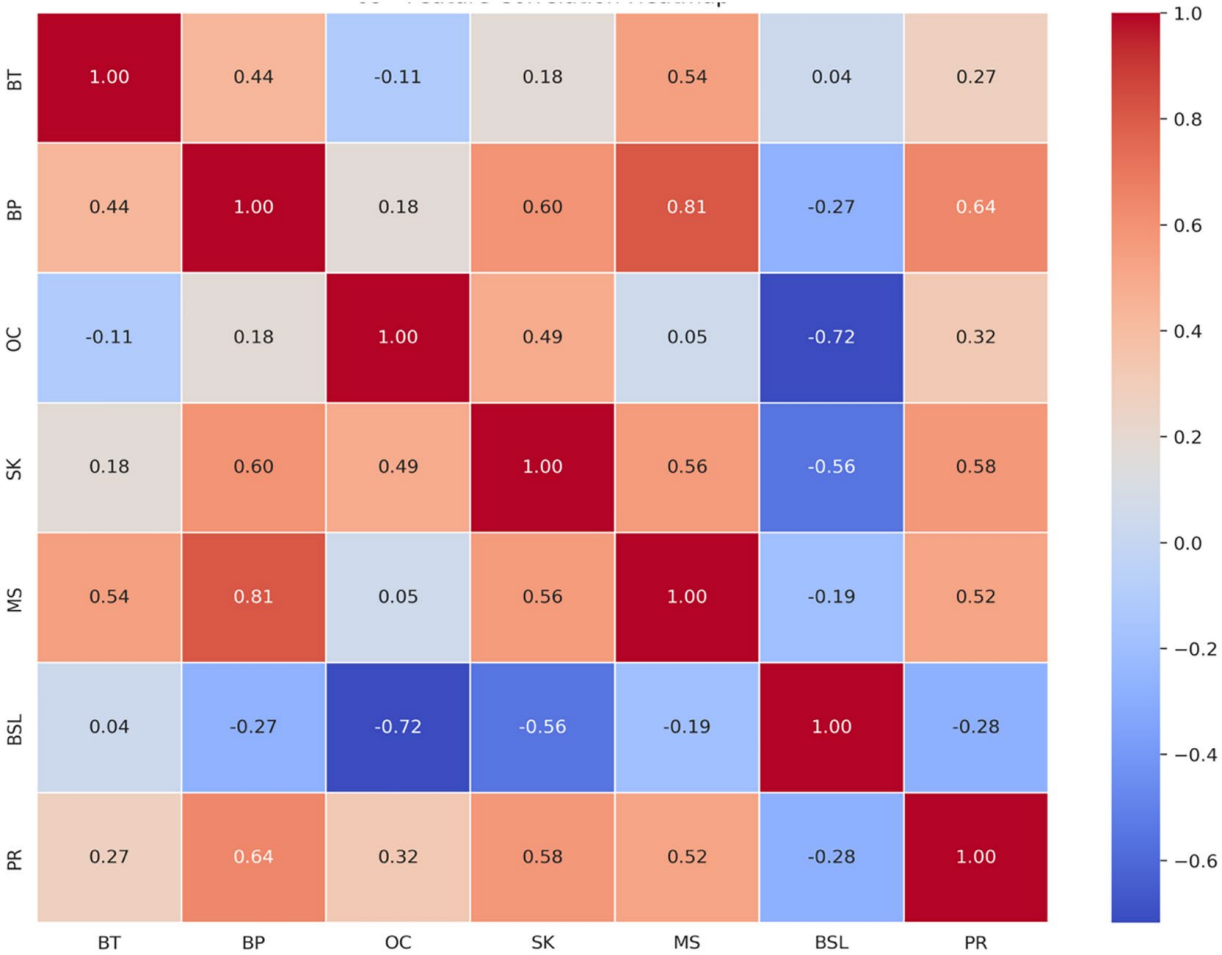
Feature co-relation of internal body parameters of SERS.

Figure 19 shows the correlation heatmap of the internal body parameters in the proposed emotion recognition system: BT (body temperature), BP (blood pressure), OC (oxygen concentration), SK (skin conductance), MS (muscle signal), BSL (blood sugar level), and PR (pulse rate). A few physiologically meaningful relationships can be noticed. Strong positive correlations are observed between BP and MS (r = 0.81), BP and PR (r = 0.64), and SK and MS (r = 0.56); this signifies that cardiovascular activities, muscle tensions, and skin responses all increase at the same time for high-intensity emotions.

Moderate positive correlations are also noticed among SK, PR, and MS, indicating complementary physiological behavior. On the contrary, BSL presents strong negative correlations with OC (r =  − 0.72) and SK (r =  − 0.56), underlining inverse physiological responses that provide discriminative information on emotion classification. Several features show weak correlations, which indicates low redundancy and preserves diversity among the features. In general, this correlation structure verifies that internal body parameter selection provides both correlated and independent information that guarantees its effectiveness in robust emotion recognition.

After the analysis of the feature correlation, the dataset that consists on these features is used to trained and test the multiple machine learning models and then evaluate the performance of these models. For an unbiased and thorough comparison, eleven top-rated classification algorithms were trained and tested on the same dataset. Each model was quantitatively evaluated by using standard performance metrics such as accuracy, precision, recall, F1-score, MCC and multi-class AUC measures. The results of this quantitative evaluation are summarized in Tabel 13.

**Fig. 20:**
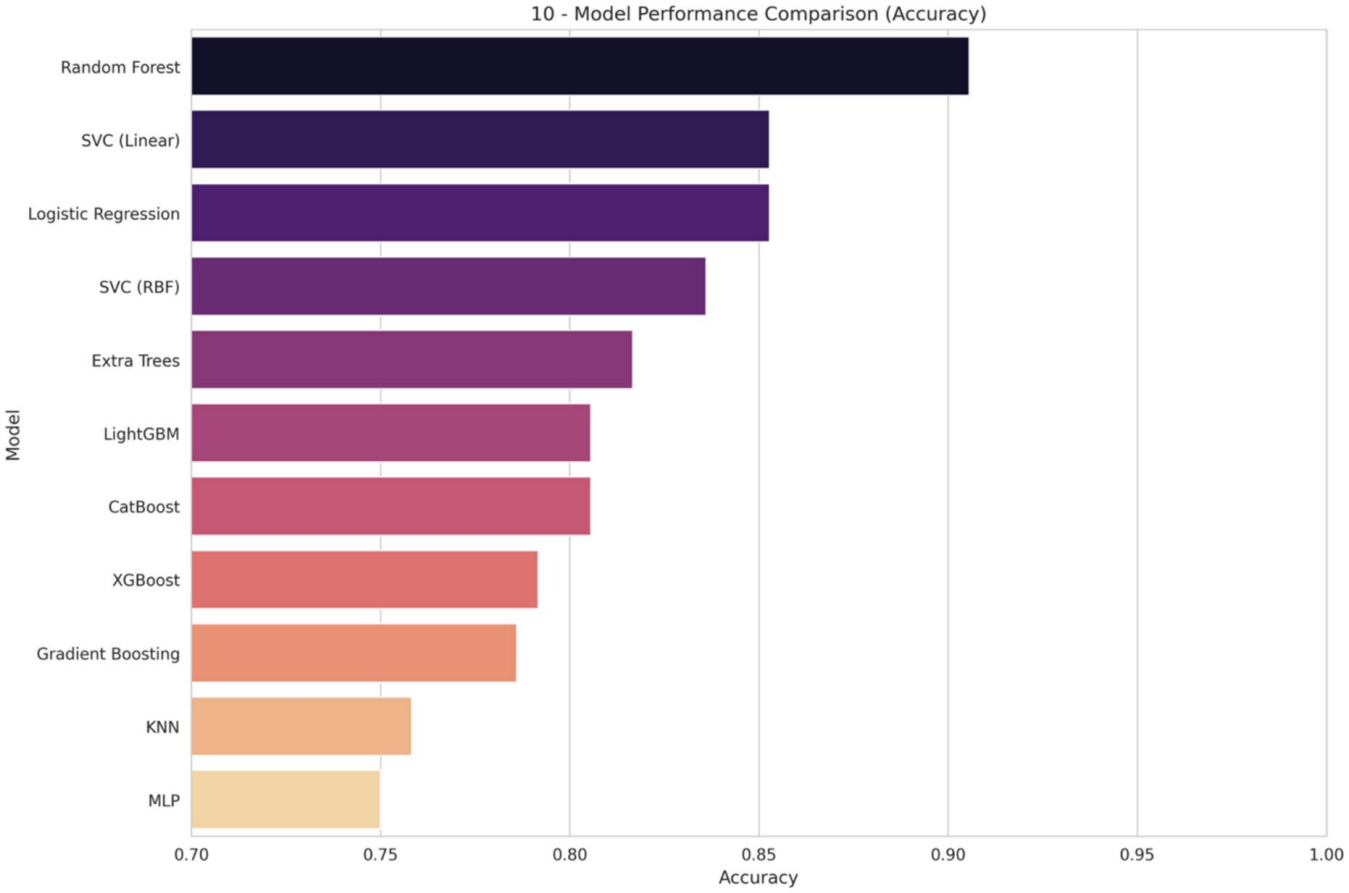
Comparison of different model of machine learning.

Table 13 summarizes the overall performance comparison of eleven machine learning models such as Logistic Regression, Multilayer Perceptron (MLP), K-Neartest Neighbors(KNN), Gradient Boosting, XGBoost,CatBoost,Light GBM, Extra Trees,SVC(RBF) and Random Forest classifiers on the proposed emotion recognition dataset in terms of weighted accuracy, precision, recall, F1-score, and multi-class AUC measure. Due to high generalization capability for all classes of emotions, the Random Forest classifier achieved the best performance with an accuracy of 90.56% as showing in Fig. 20.

The precision of random forest is 90.34%.The Support Vector Classifier model with a linear kernel, and Logistic Regression had promising results with accuracies of 85.28%, while the SVC model with RBF as the kernel had a bit lower accuracy of 83.61%. The accuracy of Extra Trees,Light GBM, CatBoost, XGBoost, and Gradient Boosting is 83.61%, 81.67%, 80.56%, 80.56%,79.17%,78.61% respectively. While the Accuracy of K-Nearest Neaighbor (KNN) and Multi-layer preceptor is 75.83% and 75.0% respectively. While the weighted precision of a Support Vector classifer (Linear) and logistic regression is 85.90% and Support Vector Classifier (RBF) is 84.26%. The precision of Extra Trees,Light GBM, CatBoost, XGBoost, Gradient Boosting, KNN and MLP is 82.35%, 81.08%, 80.99%, 78.89%, 79.67%, 76.84% and 75.49%.

In terms of precision, recall, f1-score, AUC and MCC, the RF classifier attained the maximum scores of 90.34%, 90.56%, 90.76%, 97.84% and79.25% respectively. These results show that the RF is the best-performing classifier.The second and third best-performing classifiers were the SVC and Logistic regression.

**Fig. 21:**
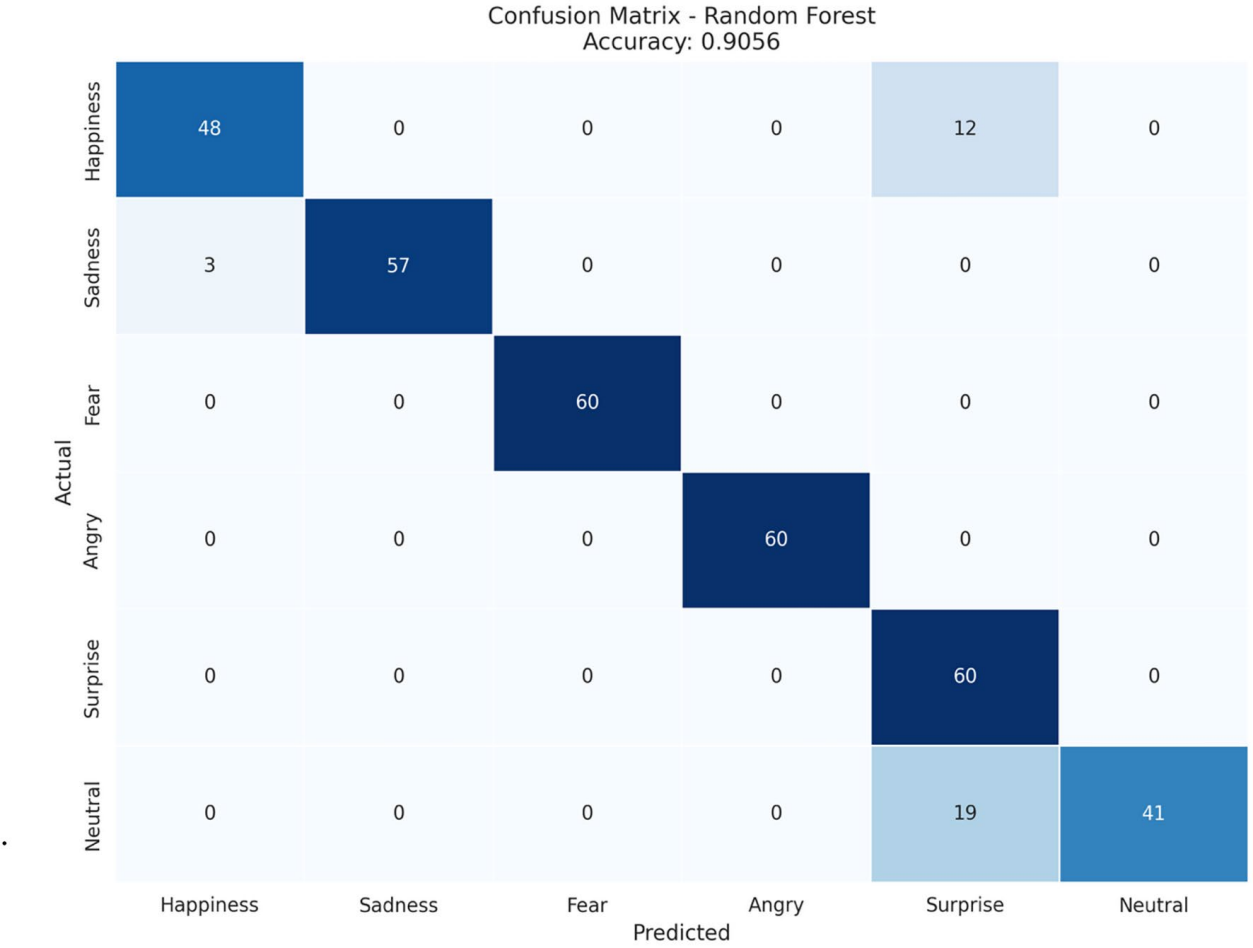
Confusion matrix of random forest model.

Figure 21 illustrates the confusion matrix of the Random Forest classifier for multi-class emotion recognition, which has attained an overall accuracy of 90.56%. Indeed, the model has done an excellent job of classifying most emotional states. For the fear, angry, and surprise emotions, perfect classification accuracy has been achieved since all the instances have been correctly predicted without any misclassification. The sadness also yields a high performance in recognition, as 57 of a total of 60 instances have been classified correctly, with only a limited number misclassified as happiness. Happiness detects 48 correct predictions, and a limited number of instances are misclassified with surprise, which indicates partial physiological response overlap between these classes because mostly these two emotion are strongly interconnected. The Neutral class shows relatively higher confusion, as 41 samples correctly classify while 19 instances have been misclassified as Surprise, indicating that neutral physiological patterns partially match those of the mild arousal state. The confusion matrix indeed verifies the robustness of the Random Forest model in distinguishing emotionally expressive states while highlighting minor ambiguities between Neutral and Surprise classes.

**Fig. 22:**
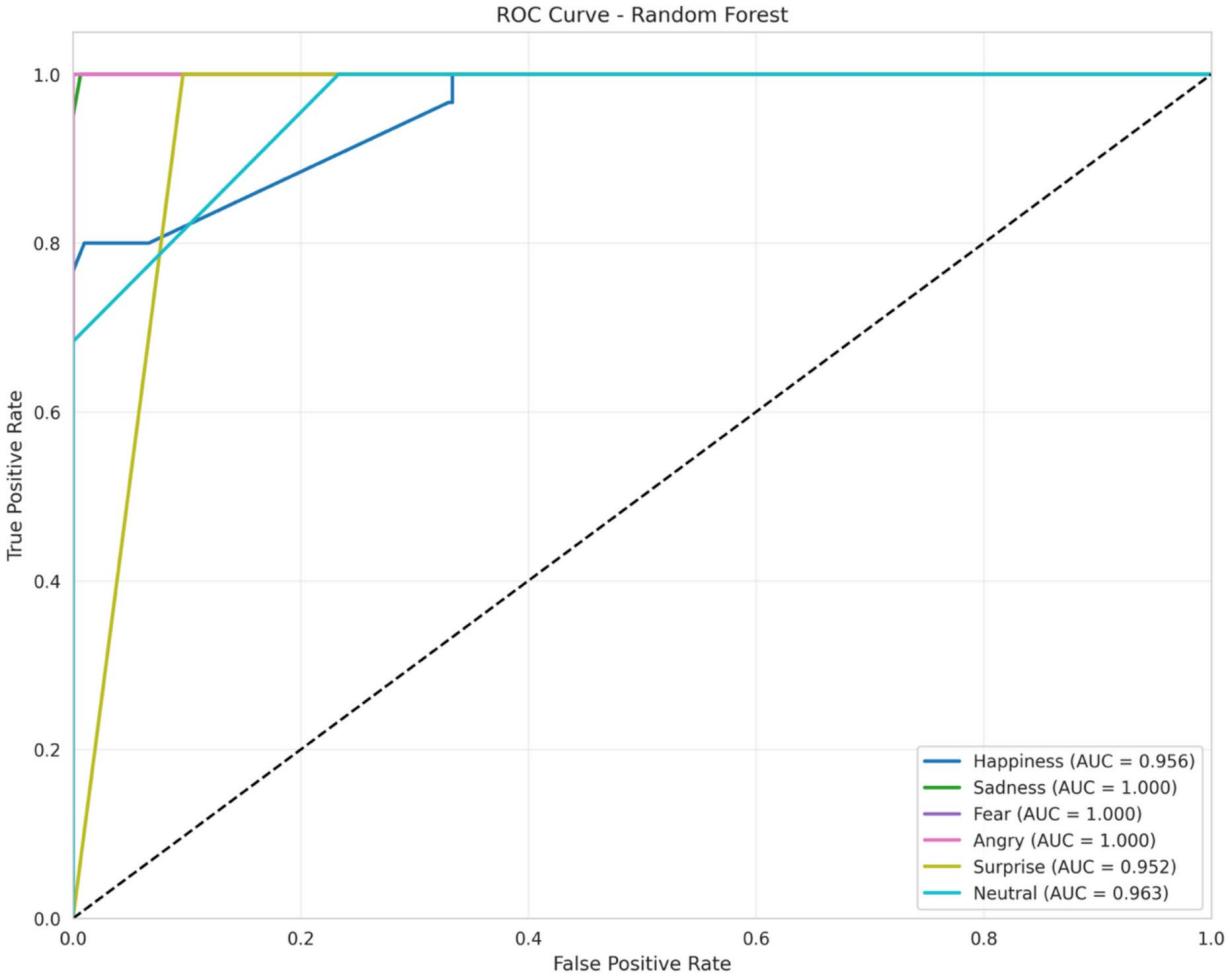
Receiver operating characteristic (ROC) curve of random forest classifier for multiclass emotion recognition.

Figure 22 illustrate the Receiver Operating Characteristic (ROC) curves for the Random Forest classification on every class of emotion. The classifier performs impressively from a discrimination perspective, with high Area Under the Curve (AUC) values for all classes. The plots indicate a condition closer to perfection for the Sadness, Fear, and Angry classes, with respective AUC values of 1.00, which means that the classification is perfect.

The remaining classes, Happiness (AUC = 0.956), Surprise (AUC = 0.952), and Neutral (AUC = 0.963), classify adequately, as the plots are close to the top-left corner of the graph, thereby proving that the Random Forest classifier efficiently distinguishes among the different states of emotion represented by the various classes.

**Fig. 23:**
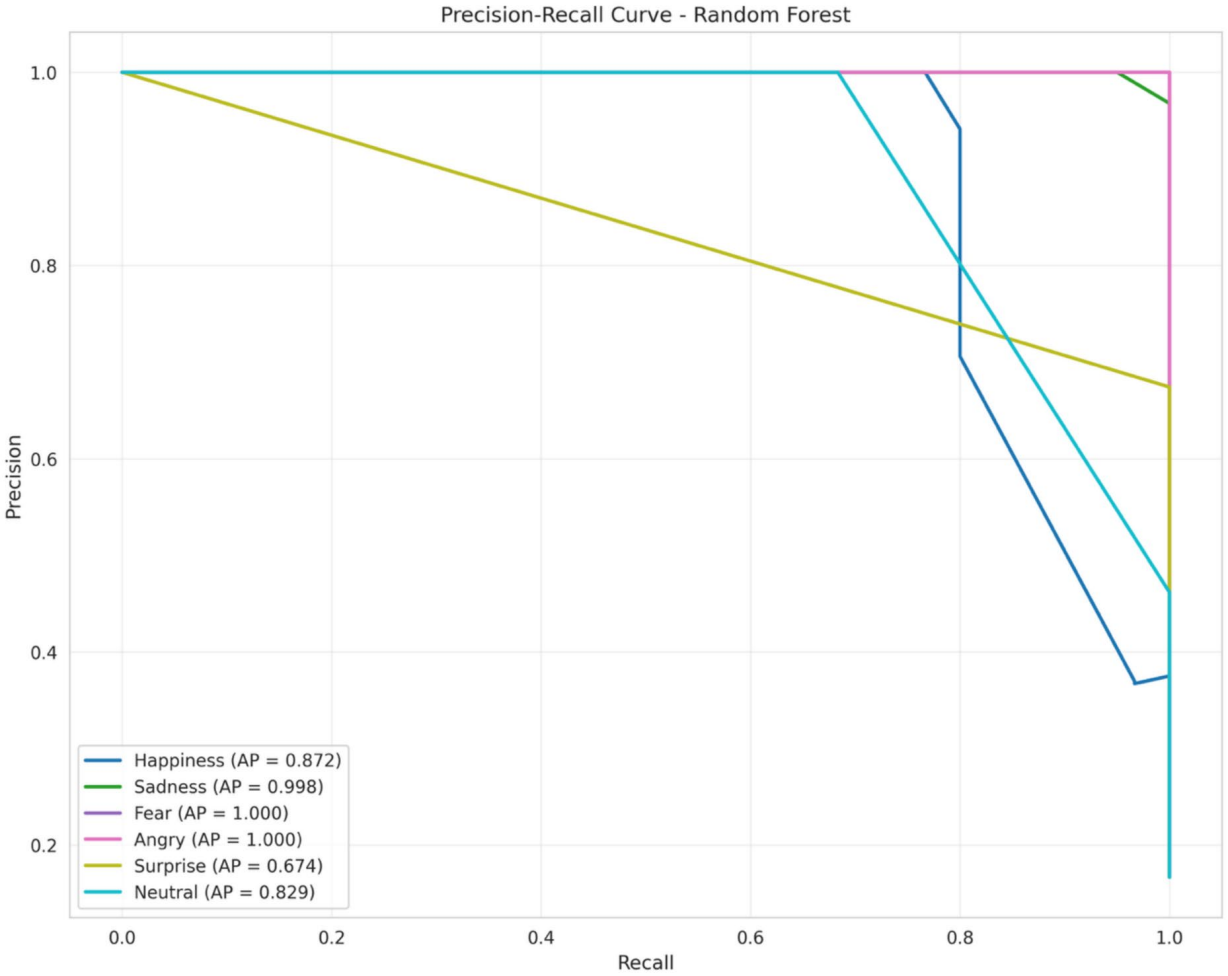
Precision recall curve of RF classifiers for multi-class emotion recognition.

Figure 23 shows the Precision-Recall Curve for the Random Forest classifier for each emotion class. It is evident that the precision-recall performance is better for almost all emotional states. Specifically, the Precision-Recall Curve for the Fear class, as well as that for the Angry class, is performing better, while that for the Sadness class is also good.

Happiness & Neutral class shows consistent performance with AP values of 0.872 & 0.829 indicating a balanced trade-off between the precision & recall. On the other hand, the class Surprise reports a relatively lower value of AP of 0.674, indicating that it is more difficult to classify, that could be correlated to their similarities in the physiological aspects.

Precision-Recall analysis validates that the Random Forest Classifier preserves a consistent prediction accuracy with respect to the classes with more stable prediction results or those that possess distinctive physiological signals for various specified classes of emotions. This observation validates the previous one with respect to the ROC analysis.

### Cross validation (K-fold) performance of random forest model

**Fig. 24:**
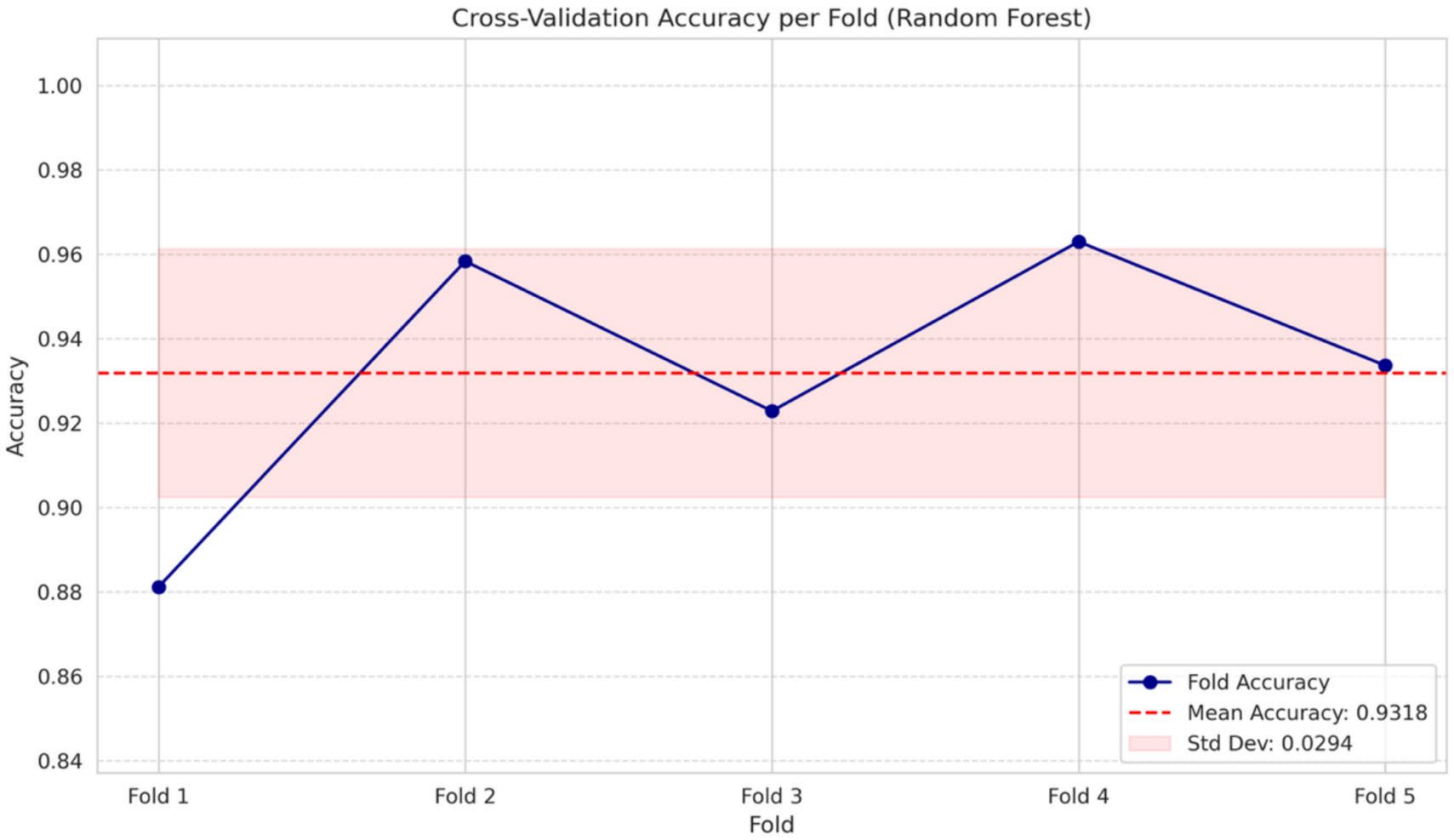
Cross validation accuracy of random forest model in SERS.

A stratified five-fold cross-validation was performed to evaluate the robustness of the Random Forest classifier. The obtained fold-wise accuracies ranged from 88.1% to 96.3%, with a mean accuracy of 93.18% and a standard deviation of 2.94% as described in Fig. 24. The low variance across folds indicates consistent model performance and robust generalization capability across different subsets of the data.

**Fig. 25:**
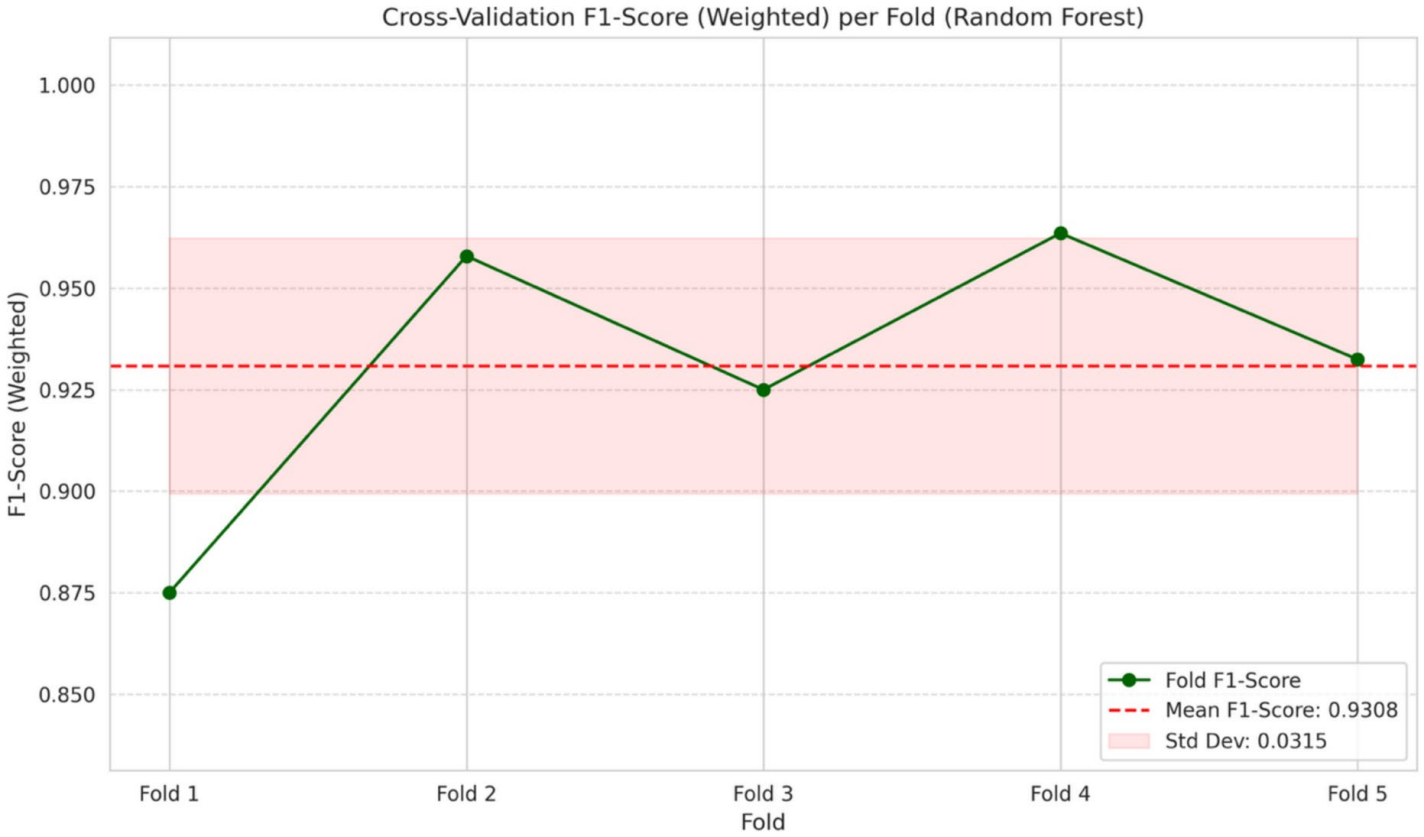
Cross validation F1-score of random FOREST model in SERS.

Figure 25 presents the weighted F1-score obtained during cross-validation. A mean F1-score of 93.08% with a standard deviation of 0.031 indicates robust classification performance across multiple emotion classes.

### External model validation using DEAP_based emotional stimuli 

To validate the performance of the proposed SERS, additional experiments using externally obtained physiological data, with 17 subjects exposed to DEAP emotional stimuli, were conducted. The Random Forestclassifier proved to be a better generalizer when tested on values that aregenerted by standard emotion induction process.

**Fig. 26:**
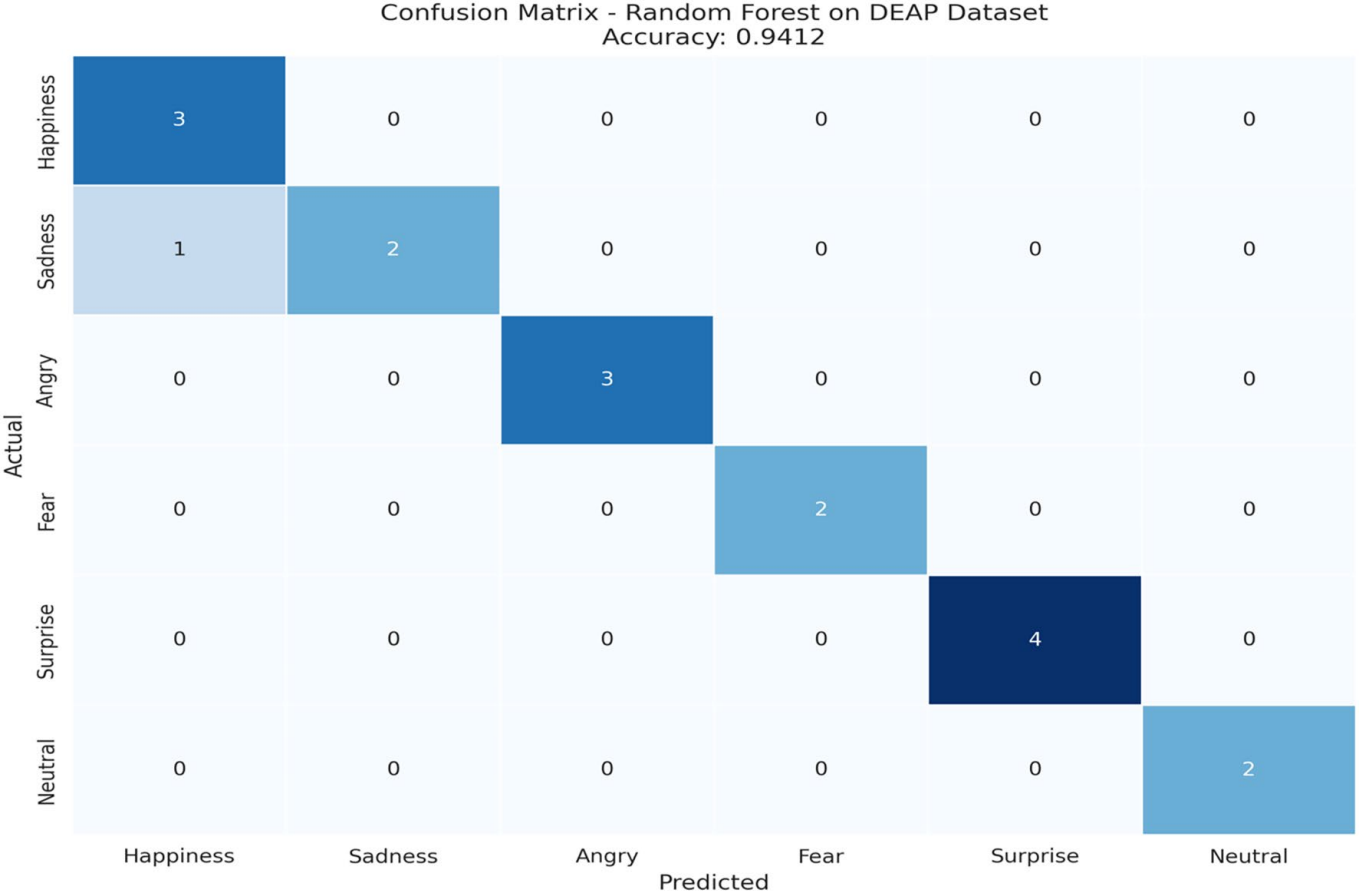
Confusion matrix of random forest on DEAP dataset.

Figure 26 describe that the confusion matrix represents a better accuracy in the classification, with little to no misclassifications among the six classes of emotions. The external validation of the model tested its accuracy with 94.12%.

Table 14 summarizes the quantitative performance of the Random Forest classifier during external validation using DEAP-based emotional stimuli. The model achieved an overall accuracy of **94.12%**, with a weighted precision of 95.59%, recall of 94.12%, F1-score of 93.95%, AUC 100% and MCC of 93.25%. The high precision indicates a low false-positive rate across emotional classes, while the balanced recall and F1-score demonstrate consistent emotion recognition performance across participants.

**Table 13:** Comparison of different classifiers of machine learning.

Model	Accuracy (%)	Precision (weighted) (%)	Recall (weighted) (%)	F1 Score (weighted) (%)	MCC (%)	AUC (OvR) (%)
Random Forest	**90.56**	**93.34**	**90.56**	**90.76**	**79.25**	**97.84**
SVC(Linear)	85.28	85.90	85.28	85.36	82.43	96.52
Logistic Regression	85.28	85.78	85.28	85.29	82.44	96.50
SVC(RBF)	83.61	84.26	83.61	83.76	80.41	97.22
Extra Trees	81.67	82.35	81.67	81.80	78.09	96.97
LightGBM	80.56	81.08	80.56	80.51	76.81	95.84
CatBoost	80.56	80.99	80.56	80.64	76.72	96.92
XGBoost	79.17	78.89	79.17	79.32	75.10	96.18
Gradient Boosting	78.61	79.67	78.61	78.92	74.42	95.28
KNN	75.83	76.84	75.83	76.16	71.07	92.49
MLP	75.00	75.49	75.00	75.04	70.07	93.30

**Table 14:** Performance metrics of Random Forest during external validation using DEAP-based emotional stimuli.

Metrics	Values (%)
Accuracy	94.12
Precision	95.59
Recall	94.12
F1-Score	93.95
AUC	100
MCC	93.25

These experiments have proved that the patterns learned from the internal dataset are applicable for the classification of the external induced emotion dataset. This consistency between the internal cross-validation experiment and the external experiment further emphasizes the robust nature of the Random Forest classifier when dealing with the variety in the physiological signals.

## Conclusion and future work

This study proposed an IoT-enabled smart emotion recognition system based on internal body parameters, such as body temperature, blood pressure, oxygen concentration, skin conductance, muscle signal, blood sugar level, and pulse rate. Dataset of internal body parameters is collected by using senors. A comprehensive feature correlation analysis was performed to show that the chosen physiological parameters have a proper balance between the correlated and independent relationships, providing substantial feature variance while minimizing redundant information. In this way, the suitability of the selected features for efficient emotion classification was justified.

Eleven machine learning models were tested to gauge the robustness of the proposed system by the standard performance metrics. In this regard, the comparative study clearly underlines that the Random Forest classifier performed better than the other models with the highest accuracy, F1-score, and AUC values showing the strong generalization capability and reliable multi-class discrimination performance. Other approaches based on ensembles and boosting showed competitive results. Conversely, instance-based and neural network approaches are much weaker.

The further class-wise validation with the confusion matrix of the Random Forest model provided evidence of its robustness, since most of the emotional states received perfect or near-perfect recognition and limited misclassifications only took place between Neutral and Surprise emotions, which can be physiologically reasonable as well. The ROC curve analysis showed results of an excellent discriminative capability within each class of emotion and even perfect AUC values for several classes, while the Precision–Recall Curve analysis further confirmed stability in that model under class-wise precision and recall trade-offs.

The metholodolgy is validated by Internal and external validation methods. The Internal validation is done by Cross Validation K-fold method and external validation is done by standard DEAP-based emotional stimuli.

Overall, the experimental results confirm that the proposed IoT-based emotion recognition framework, combined with Random Forest classification, forms an accurate, reliable, and scalable solution for multi-class emotion detection. These findings point out the potential of internal body parameters in real-time emotion recognition and give support for the potential applicability of the proposed system in monitoring health care, human–computer interaction, and affective computing applications. Future work may be directed to real-time deployment, deep learning-based hybrid models, and additional physiological signals to enhance the performance of the system further.

### Future work

Future work can further the Smart Emotion Recognition System proposed here with integration of deep learning and multimodal fusion techniques to enhance accuracy, robustness, and real-time responsiveness. Deep learning models like Convolutional Neural Networks (CNNs), Long Short-Term Memory (LSTM) networks, or Transformer-based models can be trained on bigger datasets to automatically learn intricate spatial–temporal features from biosignals. This will reduce laborious feature engineering and enable the model to pick up nuanced emotional differences between subjects.

In addition, a fusion of external parameters (e.g., facial expressions, tone of speech, or gaze direction) with existing internal physiological parameters (e.g., heart rate, blood pressure, oxygen level, body temperature, blood glucose, and muscle activity) can be used to make multimodal emotion recognition possible. A model-level fusion approach—where feature representations from internal and external modalities are fused through deep networks—can be employed to make use of complementary information from both sources. For example, CNN-LSTM hybrid networks or attentional fusion networks may be used to discover correlations between physiological signals and visual/audio features, making emotion classification more reliable across varied environments.

Further, future research should investigate real-time affect detection with wearable IoT devices and cloud computing, allowing adaptive systems to respond in real time to users’ emotional responses. The inclusion of transfer learning and cross-subject generalization methods would further enhance the scalability and relevance of the system for real-world applications like healthcare tracking, education, and human–computer interaction.

Whereas the present study provides a solid basis, there are several improvements that can be made in follow-up work:

**Larger Dataset collection**: Future studies should recruit larger and more representative populations with divergent age groups, genders, and states of health in order to enhance the model’s generalizability and quality.

**Real-time system deployment**: Designing an entire wearable system that is fully integrated, capable of real-time operation, and testable within real-world settings (i.e., classrooms, hospitals, homes) would increase the system’s practical usefulness.

**Integration with deep learning**: Deep learning techniques such as CNNs or LSTMs might be investigated to learn intricate temporal patterns in physiologic signals for enhanced accuracy.

**Multi-modal emotion detection**: Future systems can use internal body parameters in conjunction with external features like facial expression, tone of voice, or text input to create a more complete and precise emotion detection model.

**Personalized emotion models**: Physiological responses to emotions are different for individuals. Using adaptive algorithms that learn individualized emotional baselines can enhance recognition accuracy.

**Applications in health and mental wellness**: The model can be used to identify stress, anxiety, or the onset of mental illness, allowing for early intervention and care with healthcare monitoring systems.

In summary, this research provides new avenues for emotion recognition from internal signals, providing scalable solutions in healthcare, smart environments, and emotionally intelligent systems.

Although the present results are encouraging, more extensive experiments and deployment tests in naturalistic environments would be required to validate system reliability, user differences, and long-term consistency. These future endeavors would establish the applied feasibility of emotion recognition systems in envisaged applications in healthcare, education, or assistive technology, although such uses are exploratory in nature at this point.

### Limitations

Though the tested IoT-based emotion recognition system indicates promising outcomes, there are a number of limitations to be taken into account:

## Small dataset size

The training and validation dataset was small in size, which could potentially affect the generalizability and reliability of the proposed model. Future research should also make the dataset larger with more participants and a variety of demographic groups to enhance performance and reliability.

### No controlled stimuli

No standardized or controlled stimuli were used to induce emotions, so there might be inconsistencies in the acquired physiological responses.

### Restricted emotion categories

A mere selection of six primary emotions were considered. Advanced or blended emotional states were not investigated. Although this gives a good starting point, actual emotional states encountered in real-world situations are normally even more subtle and complex. Adding more emotions or compound emotional states would make the system more universally applicable in various situations.

### Offline analysis only″

The present implementation is restricted to offline analysis. Real-time functioning and response were not assessed.

### Sensor accuracy and calibration

The accuracy of internal body parameter measurements can be sensor quality and calibration dependent, influencing the reliability of input data.

### Limited external validation

Even though validation against the DEAP dataset was performed, more benchmarking against other standard datasets is required to ensure the robustness of the proposed model.

## Data Availability

The datasets used and/or analysed during the current study are available from the corresponding author on reasonable request. The data include sensitive biometric readings of human participants, and due to confidentiality agreements and ethical considerations, public sharing is restricted. However, de-identified versions can be provided upon formal request and subject to approval from the ethics committee.

## Abbreviations

SERS: Smart emotion recognition system
ML: Machine learning
IoT: Internet of Things
BP: Blood pressure
OC: Oxygen concentration
HR: Heart rate
BT: Body temperature
BSL: Blood sugar level
MS: Muscle signal
RF: Random forest
SVM: Swarm vertical machine
DT: Decision tree
KNN: Nearest neighbors node
LR: Logistic regression
XB: Gradient boost
EMG: Electric muscle signal
